# Polymeric Nanoparticles for the Treatment of Malignant Gliomas

**DOI:** 10.3390/cancers12010175

**Published:** 2020-01-10

**Authors:** Basant Salah Mahmoud, Ali Hamod AlAmri, Christopher McConville

**Affiliations:** 1 College of Medical and Dental Sciences, School of Pharmacy, University of Birmingham, Birmingham B15 2TT, UK; BSM465@student.bham.ac.uk (B.S.M.); or; 2 Hormones Department, Medical Research Division, National Research Centre, El Buhouth St., Dokki, Cairo 12622, Egypt; 3 College of Pharmacy, King Khalid University, Abha 62585, Saudi Arabia

**Keywords:** brain tumours, glioma, blood brain barrier, drug delivery, nanomedicine, polymeric nanoparticles, PEGylation

## Abstract

Malignant gliomas are one of the deadliest forms of brain cancer and despite advancements in treatment, patient prognosis remains poor, with an average survival of 15 months. Treatment using conventional chemotherapy does not deliver the required drug dose to the tumour site, owing to insufficient blood brain barrier (BBB) penetration, especially by hydrophilic drugs. Additionally, low molecular weight drugs cannot achieve specific accumulation in cancerous tissues and are characterized by a short circulation half-life. Nanoparticles can be designed to cross the BBB and deliver their drugs within the brain, thus improving their effectiveness for treatment when compared to administration of the free drug. The efficacy of nanoparticles can be enhanced by surface PEGylation to allow more specificity towards tumour receptors. This review will provide an overview of the different therapeutic strategies for the treatment of malignant gliomas, risk factors entailing them as well as the latest developments for brain drug delivery. It will also address the potential of polymeric nanoparticles in the treatment of malignant gliomas, including the importance of their coating and functionalization on their ability to cross the BBB and the chemistry underlying that.

## 1. Introduction

In the past decade, there has been a great development in medicine and cancer treatment. However, cancer remains a challenging health issue owing to its complicated nature [1,2]. The number of cancer cases is expected to rise to 27.5 million in 2040, as stated by the American Cancer Society [1]. Among the most troublesome malignant cancers are the primary brain cancers that can rarely be cured, with a 5-year overall survival of only 35%. Gliomas count as the most common form of malignant primary brain tumours in adults [3].

The speed and ability to infiltrate and metastasize to nearby brain tissues are the main factors that determine if glioma cells are of low grade (WHO I and II) or high grade (WHO III and IV) [4]. Gliomas have the ability to infiltrate to surrounding tissue and their margins are difficult to determine. This results in conventional treatment approaches being insufficient to produce a curative outcome. Also, the difficulty in achieving successful therapeutic approaches is caused by the physical and chemical barriers that exist, hampering drugs from reaching tumour sites [5,6,7,8]. The blood brain barrier (BBB) and blood brain tumour barrier (BBTB) represent the main barriers that stop drugs from entering the brain unless they possess certain characteristics. Also, the multipotent stem cells that give rise to glioma cells, have the ability to self-renew and are responsible for glioma recurrence [9]. Efforts have been extended towards overcoming physical hurdles by developing techniques that deliver therapeutics to the brain, however, most of these approaches are invasive and fraught with serious side effects, so curative measures should not be only be based on extending survival, but also towards improving the quality of life of patients by reducing side effects. Among the advanced therapeutic strategies is using polymeric nanoparticles for drug delivery and targeting. As will be discussed later in this review, in vitro and in vivo studies have reported promising results for drug loaded nanoparticles targeted to gliomas. Therefore, more efforts should be made towards the betterment of these nanomedicines in terms of improving their loading efficiencies, coating and ability to target gliomas.

## 2. High Grade Gliomas

Primary malignant brain tumours in adults are mostly gliomas, 75% of which are high grade gliomas (HGG) diagnosed in the central nervous system (CNS). The rate of incidence is 3 to 5 per 100,000 every year, afflicting mostly men. HGG can occur at any age, however, they mainly occur in the 5th and 6th decades of life [10].

Bailey and Cushing developed the seminal system for defining the morphology of glial tumours in the 1920s which is based on the glia stage of growth [11]. This system was used by the WHO in 2000 to classify Gliomas based on their morphology [12]. Gliomas were further stratified based on genetic and molecular factors that were recognized and reported by the WHO in 2016. These factors include isocitrate dehydrogenase (IDH) mutation status, the co-deletion status of 1p/19q and the mutation status of alpha thalassemia/mental retardation syndrome X-linked protein/gene (ATRX) [12]. According to the 2016 WHO classification, grade III tumours which include anaplastic astrocytoma, anaplastic oligodendroglioma and mixed anaplastic oligoastrocytoma are among HGGs, in addition to grade IV glioblastoma (GBM) [12]. GBM has the highest incident rate among HGGs with up to 60–70% of cases being a GBM. This is followed by anaplastic astrocytoma which makes up 10–15% of cases. Whereas, anaplastic oligodendrogliomas and anaplastic oligoastrocytomas have the least frequent incidence rate of only 10% [13]. Some other types of malignant gliomas, such as the WHO grade III gliomas, anaplastic ganglioglioma, anaplastic pilocytic astrocytoma and anaplastic pleomorphic xanthoastrocytoma and the grade IV gliomas, giant cell and small cell GBM, epithelioid GBM, and gliosarcoma are not very common. The main cause for HGGs is still enigmatic, with ionizing radiation only identified as a possible risk factor [14].

## 3. Treatment

### 3.1. Surgical Resection

The site, grade and morphology of the tumour will determine if complete surgical resection of the tumour can be achieved. Patients with high grade tumours require near complete resection in order to reduce the burden of the tumour and pressure inside the skull, which in turn improves the survival rate [15,16,17,18]. GBM cannot be fully cured with surgical resection as it is invasive in nature and 80% of cases result in relapse within 2 to 3 cm of the original tumour margin [19].

### 3.2. Radiation Treatment

Radiation therapy (RT) can be administered internally or externally and is considered the standard treatment protocol for HGGs [20]. The standard treatment using external RT involves 25 to 35 treatments on a daily basis for (5–7) weeks. Several factors control the total radiation dose to be administered which are: tumour site, grade, histology and the extent of resection [21]. A randomized trial conducted in the 1970s reported that whole brain irradiation with 60 Gy after surgical resection enhanced survival for patients suffering from HGG. This resulted in RT as being a standard therapy following tumour eradication for HGG [22]. On the other hand, studies that investigated the difference between partial and whole brain irradiation for HGG treatment proved that whole brain irradiation did not provide extra benefit compared to partial irradiation of the brain [23]. However, there was an improvement in the delineation accuracy that was achieved employing tomography and magnetic resonance for maintaining a partial irradiation of the brain for HGG cases [24]. The developments that have occurred in imaging and RT have enabled irradiation of tumour regions with higher doses while reducing the volume of normal brain tissue exposed to irradiation. Therefore, involved field RT was granted acceptance as the standard of care for HGGs. However, the issue of RT delivery for smaller surface areas remains debatable with efforts towards targeting infiltrating tumour cells [25]. Some techniques that have been recognized for providing a more targeted irradiation towards tumour tissues include fixed field intensity modulated RT (IMRT), dynamic arc IMRT, volumetric-modulated arc therapy (VMAT) and stereotactic radiosurgery (SRS). These techniques were reported to provide a more focused approach towards affected tissues while reducing toxicity to normal tissues [26]. SRS is used to treat recurrent GBM and is used as a complementary treatment after external beam RT. However, the use of SRS for the treatment of recently diagnosed malignant gliomas is still under review [21]. Another approach for RT is interstitial RT or brachytherapy where radioactive material is implanted inside the tumour with the use of surgery. Proton therapy could also aid in targeting affected areas and may be used instead of photon irradiation [26]. RT is associated with some limitations including necrosis of normal brain tissue, neuronal damage and radiation resistance of tumour cells [19].

### 3.3. Chemotherapy

The chemotherapeutic drug temozolomide (TMZ) is used in combination with RT in patients with HGG in order to improve the survival rate of patients. This protocol, known as the Stupp protocol, demonstrated a median survival rate of 14.6 month compared to 12.1 months (when RT alone was administered) in a phase III clinical trial. TMZ is initially administered at a daily dose of 75 mg/m^2^ for 6 weeks, with a 1-month rest period upon the completion of RT treatment. TMZ treatment begins again with a daily dose of 150 mg/m^2^ for 5 days in the first month. If this dose can be withstood by the patient, a higher daily dose of 200 mg/m^2^ is administered for 5 consecutive days each month until the end of the treatment period. The stupp protocol administers the TMZ therapy for 6 months following RT [27]. Synergistic effect of combined therapy using RT and adjuvant chemotherapy with TMZ continued over a 5-year follow up treatment period. Also, the inclusion of TMZ was more beneficial for patients with a methylated gene promoter that encodes O-6-methylguanine-DNA methyltransferase (MGMT). This in turn had resulted in identifying MGMT as the first biomarker in brain tumours to help anticipate the responsiveness to the TMZ treatment and selection of patients accordingly [28]. However, MGMT is unreliable for patients who do not have a methylated promoter of MGMT except for elderly GBM patients [29]. Some other chemotherapeutic drugs have shown efficacy against recurrent malignant gliomas. These drugs are methylating agents such as irinotecan or those that target the vascular endothelial growth factor such as bevacizumab. Other chemotherapy drugs such as gefitinib, erlotinib and imatinib target the epidermal and platelet-derived growth factor receptors [30].

Among the latest therapeutic approaches is a device named Optune^®^ that received approval by the U.S. Food and Drug Administration (FDA) in October 2015. This device allows for the delivery of electric fields that enable the treatment of tumours by interrupting the division of cells causing cell death. It is used as an adjuvant therapy with TMZ following surgical resection and is the standard of care for adults that have been recently diagnosed with supratentorial GBM. The Optune^®^ treatment regimen alongside TMZ increased survival from 4 to 7 months when compared to treatment with TMZ alone [31]. Optune^®^ was initially approved by the FDA in 2011 as a single treatment for recurrent GBM. Optune^®^ is alternatively used as a treatment for primary GBM after surgery and RT have been shown to be ineffective. A randomized clinical trial exhibited similar rate of survival and less side effects with a significant reduction in the infectious, gastrointestinal and hematologic complications for the Optune^®^ treatment group compared to the standard chemotherapy group [32]. The reduced side effects and practicality of use encouraged the National Comprehensive Cancer Network (NCCN) to include Optune^®^ as a treatment for recurrent GBM [33].

Other treatment approaches such as radioimmunotherapy, iodine-125 brachytherapy, hyperfractionation and SRS have been investigated for their ability to localise treatment and protect normal brain tissues. However, none have been shown to improve survival rates [34]. Therefore, it was concluded that chemotherapy used concomitantly and adjuvantly with RT is the standard of care in current application for GBM patients until more effective treatments become available [28].

## 4. Postoperative Treatment

Follow up is necessary to monitor the postoperative complications and control the disease symptoms, which include seizures, cerebral edema, turbulences in the gastrointestinal tract, osteoporosis, venous thromboembolism, dysfunction of the cognitive abilities and mood deterioration [35]. Magnetic Resonance Imaging (MRI) scans are used to monitor the size of the tumour and preformed 3 days following the surgical operation to determine how much of the tumour was eradicated. Steroids are administered to patients suffering vasogenic edema. However, treatments based on steroids are associated with adverse side effects such as myopathy [36] which affects 10% of patients with HGGs with an increased incidence rate in elderly patients that are administered corticosteroids for long time periods [37]. Also, patients become vulnerable to mental impairment, hyperglycemic and gastrointestinal complications, in addition to opportunistic bacterial infections such as *Pneumocystis jiroveci*. Dexamethasone is a more favourable corticosteroid as it is lower in activity and upon its discontinuation, myopathy can be reversed [37,38]. Patients with GBM and CNS lymphoma are more susceptible to venous thromboembolism, especially after craniotomy. Treatment using warfarin or heparin of low molecular weight is more favourable than vena cava filters in controlling the anticoagulation and reducing the complications. Levetiracetam is usually administered to patients suffering seizures due to its low toxicity and the fact that it does not interact with the chemotherapy drugs [38].

## 5. Prognosis

Patient prognosis remains depressing with a 15-month median survival, despite the advancements in surgical resection of the tumours [39]. Anaplastic astrocytoma afflicted patients average between 2 to 3 years survival [40]. The best prognosis is shown in cases suffering anaplastic oligodendroglioma leaving them with expected average survival of 12 to 15 years [41]. The prognostic factors involve: the extent of tumour resected, age of the patient and the Karnofsky Performance Status. Younger age and higher performance status could imply longer survival. Negative results have been linked to tumours larger than 5 to 6 cm [42]. Surgically curable tumours such as those that arise in the cerebrum or cerebellum have a better prognosis than those that arise in the brainstem or diencephalon [43].

## 6. Glioblastoma Multiforme

Glioblastoma multiforme (GBM) is a malignant tumour that arises in the brain and is known to be the most common form of brain tumours. It represents 16% of the tumours that originate in the brain and CNS [39]. GBM has an incidence rate of 3.2 per 100,000 population [44,45]. GBMs are mainly located in the brain but can also occur in the brain stem, cerebellum and spinal cord. Moreover, the four lobes of the brain (frontal, temporal, parietal and occipital) represent the main sites for the development of the primary gliomas, with an overall incidence rate of 61% and individual incidence rate of 25%, 20%, 13% and 3%, respectively [46]. Initially, glial cells were thought to be the only source of GBMs, however, it has been shown that several types of cells possessing the characteristics of neural stem cells, could give rise to GBMs. These cells vary in their differentiation stage where they start out as stem cells then give rise to neurons and glia. This is accompanied with changes in their phenotype, mainly caused by the variation in their signaling pathways instead of the differences in the origin of the cell type [47]. The average age in which GBMs develop is 64 [39]. Nevertheless, GBMs can develop at any age including children. Men are more likely to develop a GBM than women with a rate of 1.6 for every woman. Caucasians are also more likely to be stricken with the disease than other ethnicities [42]. GBMs can vary in their classification, for example a GBM would be classed as primary or de novo in origin if it has developed without a defined precursor. If a GBM develops from a transformed low-grade tumour it will be termed secondary. Most GBMs are primary in nature and mainly afflict the elderly, who have a poorer prognosis than their counterparts who develop secondary GBM [48]. GBMs can be further classified into four subtypes which are, classical, pro-neural, neural, and mesenchymal. Each subtype varies in its mode of development and survival [49,50]. GBMs are invasive in nature and are difficult to completely remove by surgical resection. They often exist in sensitive areas of the brain which mainly control speech, movement or the senses. The tumour cells also possess the ability to infiltrate and remain in areas that surround the brain, which leads to further disease recurrence [48].

### Current Management

The current treatment regimen for GBM is the Stupp protocol which was discussed earlier as the standard of care for glioma management [28]. The main action of TMZ involves methylation of the DNA at the N7 and O6 positions on guanine which halts the DNA mismatch repair mechanism resulting in DNA nicks that halts the cell cycle at the G2-M level, causing apoptosis. However, the elevated level of MGMT activity which functions by protecting tumour cells against chemotherapeutic agents can negatively impact the TMZ response. Moreover, TMZ, is reported to be fraught with deleterious complications such as hematological issues, fatigue and susceptibility to infections [51]. Another chemotherapeutic agent used to treat GBM is the Gliadel^®^ Wafer, which is a disc shaped 200 mg wafer made of biodegradable copolymers that contain 3.85% *w/w* of the alkylating agent bis-chloroethylnitrosourea (carmustine, also known as BCNU). Carmustine was initially approved by the FDA as a potent antineoplastic agent for the treatment of GBM by intravenous administration [52]. Gliadel^®^ is used for local administration of carmustine, with up to 8 discs placed into the resection cavity during surgery. After treatment with Gliadel^®^ Wafers, the median survival in a group of patients with malignant glioma (95% of which was GBM) was 42 weeks, eight patients survived one year, and four patients survived more than 18 months. Local treatment allows the chemotherapy to be concentrated at the site of the tumour while avoiding systemic side effects. However, patients suffered perioperative infections, seizures and required addition steroid treatment [53]. Moreover, the drug penetration into tissues after diffusion from the implants does not exceed 1mm which limits its efficacy [54].

In summary, the drawback of these treatments is that they are associated with serious unwanted side effects in addition to the development of resistance, limiting their efficacy. Some patients do not respond to the TMZ or BCNU, therefore, there has been a second line of drugs developed which include carboplatin, oxaliplatin, etoposide and irinotecan. Additional chemotherapeutic agents for GBM include anti-angiogenic agents like anti-VEGF monoclonal antibodies (bevacizumab), anti-FGF antibodies, monoclonal antibodies targeting EGFR (erlotinib and gefitinib) and tyrosine kinase inhibitors [19,55,56,57]. Despite developments in tumour diagnosis and treatment using RT and concomitant chemotherapy with TMZ, nearly all GBM patients experience tumour recurrence. 

## 7. The Blood Brain Barrier

One of the main limitations in the systemic treatment of malignant gliomas is the presence of the BBB, which is a complex structure that comprises endothelial cells, pericytes, astroglia and perivascular mast cells and acts as a barrier to most cells, pathogens and drugs circulating in the blood. The BBB is compact in nature due to the presence of tight junctions between the endothelial cells of the vascular layer that are closely stuck together. The BBB surrounds both the brain and spinal cord capillaries and its compactness halts small molecules and ions from passing through the BBB and into the brain. The tightness of the BBB stops integral membrane proteins from moving between the apical and basolateral membranes of the cell, thus protecting the cell membrane from loss of function [58,59,60].

The tight junctions of the BBB have three fundamental proteins which are occludin, claudins, and junctional adhesion molecules. Occludin and claudins form the pillar of junction strands. Whereas, when there is an immunologic response in the brain, the junctional adhesion molecules function in the transport of lymphocytes, neutrophils, and dendritic cells from the vascular system. The tight endothelial junctions and adherens junctions are made of cadherins and catenin proteins that are responsible for the adherence of the BBB endothelial cells, forming a transelectrical resistance >1500 Ω cm^2^. Although the BBB acts as a physical barrier, it still regulates the transport of metabolic molecules to the brain for nutrition. Small molecules such as glucose or amino acids have specific transporters that convey them to the brain. While, macromolecules such as cytokines and neurotrophils enter the brain by receptor mediated endocytosis [61,62].

The BBB limits the passage of chemotherapeutic drugs with only low molecular weight, electrically neutral, hydrophobic drugs able to cross the BBB with a preference towards molecular weight less than 500 Da and lipophilicity expressed in log *P* as (2–3) [63]. Most chemotherapeutic drugs are large, ionically charged, hydrophilic molecules and thus cannot easily cross the BBB at the levels required for therapeutic effect, which means a large systemic dose is required. For example, irinotecan hydrochloride, which is a potent anionic chemotherapy drug, possesses a molecular weight of 623.1 Da and is hydrophilic in nature, therefore it will face difficulty crossing the BBB and accumulating in the tumour in its initial administered dose. Even if the drug crosses the BBB, it can very quickly diffuse back making it difficult to obtain constant drug levels in the brain after systemic administration.

## 8. Drug Delivery to the Brain

Two strategies, crossing the BBB and bypassing the BBB, are currently used for the delivery of drugs to the brain. Crossing the BBB can take place via six main pathways: paracellular transport, passive transcellular diffusion, carrier-mediated transport (CMT), receptor-mediated transcytosis (RMT), adsorptive-mediated transcytosis (AMT), and cell- mediated transport. The normal physiology of the BBB does not afford paracellular permeability [64]. However, it can take place when the BBB is compromised in CNS disorders such as GBM, which could facilitate drug delivery to the brain [65]. Transmembrane diffusion allows for the intake of most of the compounds based on their molecular weight and lipid solubility. Influx and efflux transporters facilitate mediated transport, which relies on protein carriers that bind solutes and transport them from the luminal side of the BBB to the other side of the membrane via passive or active transport mechanism [51]. Among the influx transporters are l-type amino acid transporter (LAT1), glucose transporter (GLUT1), monocarboxylate lactate transporter (MCT1), cationic amino acid transporter (CAT1), choline transporter (ChT), sodium-coupled glucose transporters (SGLTs). Influx transporters facilitate particle uptake by the BBB. Efflux transporters, on the other hand, mediate molecules exclusion form the BBB. These transporters are like p-glycoprotein (P-gp), peptide transport system-6 (PTS-6), and breast cancer resistant protein (BCRP) [66,67,68,69]. RMT involves the uptake of macromolecules by clathrin-mediated or caveolin-mediated endocytosis. This route has been previously used to deliver both free drugs and nanoparticles into the brain [70]. RMT is the route by which actively targeted drugs are internalized. Receptors expressed on the surface of cells are recognized and bound by complementary ligands coating the drug loaded nanoparticles and this complex structure enters the cell in vesicles coated with clathrin. This process is 1000 times more efficient than pinocytosis [71]. The size of clathrin-coated vesicles is determined by the size of the drug delivery vehicle which they carry [72]. Several receptors aid in transporting compounds across the BBB, such as the insulin receptor, low-density lipoprotein (LDL) receptor, transferrin receptor, neonatal Fc receptor and leptin receptor [73,74,75,76,77]. Whether internalization occurs by pinocytosis or RMT, the cargo is delivered to the early endosome, which is of slightly acidic pH (6–6.8). The endosome has a sorting function, where it either allows the recycling of molecules back to the plasma membrane or sends them to late endosome and lysosome for degradation [78]. Whereas, caveolin-mediated endocytosis forms caveolae which are invaginations in the plasma membrane that take the shape of small flasks that engulf large molecules and transport them internally. AMT relies on the electrostatic interaction between positively charged substances and the plasma membrane, leading to internalization of molecules followed by their transport across the BBB. This pathway could be exploited by developing drugs or nanoparticles with positive charges or by conjugating the drug or nanoparticles with a positively charged ligand [79,80]. CMT exploits the natural mechanism involved in inflammation with drugs and nanoparticles engulfed by immune cells such as monocytes and macrophages [81,82]. In diseases such as neuroinflammation, or GBM, immune cells such as leukocytes are transported towards the brain parenchyma by chemotaxis and diapedesis processes. This process could be exploited in designing drugs or nanoparticles that can be phagocytosed by leukocytes and thus transported into the brain. The efficacy of free drugs and nanoparticles delivered by this natural mechanism, also known as the Trojan Horse mechanism has been shown to increase. This mechanism allows for larger sized particles to enter the brain, however, their larger size can result in increased toxicity [83,84,85]. There are a number of options for bypassing the BBB. Intracerebroventricular (ICV) administration is performed through an invasive procedure of skull penetration and drug injection directly into the brain. An implantable reservoir or a pump is used to introduce the drug through an outlet catheter. The pump allows for a constant drug supply at high concentrations. The ICV process is extremely invasive and can lead to infections and increased intracranial pressure [86]. Intracerebral/intraparenchymal administration involves the delivery of drugs directly into brain tissue either via stereotactic injection or by formulating into an implant that can be implanted during resection surgery (i.e., Gliadel^®^) or via stereotactic surgery. The issue with this type of delivery is that drug diffusion occurs slowly and allows drug to travel only 2 mm from the injection/implantation site [86]. Convection Enhanced Delivery (CED) is a slightly less invasive surgical procedure where catheters are placed inside the interstitial space of the brain parenchyma. A drug solution is administered into the brain under a positive pressure gradient using a pump, leading to a higher distribution volume compared to intracerebral/intraparenchymal administration [86]. This procedure is still, however, invasive in nature and could subject patients to the risk of infections, tissue injury and air bubbles. Furthermore, due to the high pressure used, the drug solution could leak into sensitive areas of the brain, such as subarachnoid space [86]. Intrathecal administration is considered one of the least invasive procedures, where drugs are injected into the subarachnoid space of the spinal cord via a lumbar puncture where they reach the CNS parenchyma in the cerebral spinal fluid [87]. However, possible side effects such as infections and adverse immune response could occur due to this technique [87]. In addition, although the ICV and intrathecal techniques can bypass the BBB and cerebrospinal fluid (CSF) hurdles, there remain the ependymal cell layer and glial cells which come in the way between the CSF and the brain parenchyma limiting the efficacy of drug diffusion to reach the brain parenchyma via these techniques [88]. Intratympanic administration employs the route of the middle ear to administer drugs, which are transported via pinocytosis, eventually reaching the brain where they bypass the labyrinthine barrier (BLB) which is similar to the BBB. This route can be suitable for therapeutics up to 1 µm size [89]. Poly(d,l-lactide-co-glycolide) (PLGA) nanoparticles were used to administer drugs via this route with promising efficacy [90]. Intranasal delivery is a non-invasive route of administration for bypassing of the BBB through spraying drugs into the nasal cavity, where they diffuse extracellularly or via convection. Another route is through olfactory sensory neuron termed intraneuronal transport or through trigeminal nerve, termed intraneuronal transport [91]. The intranasal route is beneficial in terms of being convenient to patients, allowing for rapid absorption and avoiding first pass metabolism [92]. Some other methods for crossing the BBB have been investigated, most of which are invasive in nature, such as osmotic opening of the BBB [93]. Other non-invasive methods have also been investigated, for example, the Trojan Horse technology which relies on coupling drugs to genetically engineered proteins that can cross the BBB by receptor mediated transport processes [94]. Such methods are also fraught with side effects and thus alternative more effective and less toxic methods for delivery of drugs to the brain are needed in order to improve the treatment of brain tumours [95].

## 9. Nanomedicine: A Non-Invasive Approach towards a Better Quality of Life

Nanotechnology has provided us with a promising tool that can be used to enhance the uptake of drugs across the BBB [96,97]. This is because nanoparticles have the ability to be loaded with therapeutic agents and functionalized with multiple ligands that enable targeting and crossing of the BBB. In this case, the ability to cross the BBB will not be dependent on the structure of the drug, which cannot readily be altered, but on the physicochemical properties of the nanoparticles, which can be altered. Nanoparticles are proposed to perform their action in delivering drugs across BBB by concentrating the drug inside or at the surface of the BBB, which will result in a high concentration gradient between blood and brain, encouraging passive diffusion of the drug into the brain [98].

Nanoparticles have the ability to diffuse into the leaky vasculature of tumour tissues by the enhanced permeability and retention effect (EPR). This cancer-specific attribute is characterized by poor lymphatic drainage allowing the accumulation of nanoparticles to reach concentrations much higher than their concentrations in plasma [99]. The effective treatment of GBM can be accomplished by achieving three main goals: (1) Improving the ability of chemotherapeutic agents to cross the BBB, penetrate into brain tissue reaching the tumour tissue at therapeutic concentrations (2) Avoiding or reducing side effects and (3) Sustaining therapeutic concentrations of the drugs at the site of the tumour, increasing their half-life and avoiding rapid clearance [99].

## 10. Physicochemical Properties of Nanoparticles

The different physicochemical properties of nanoparticles such as the particle size, surface charge, hydrophobicity and coating material have an impact on the targeting process. They also impact the interaction of particles with the cell membrane and passage through biological body barriers [100]. Size is an important factor that allows transport of nanoparticles in the blood stream and enables delivery of nanoparticles to the site of the tumour. Small sized nanoparticles can easily reach the leaky blood vessels of the tumour, however, they can extravasate into the normal tissues [101]. Therefore, optimization of nanoparticles size can enhance their uptake into tumour tissues. The shape of nanoparticles is also of importance as it influences the fluid dynamics and thus particle uptake. The current trend is towards using spherical nanoparticles owing to the ease in their synthesis and application [102]. In addition, the stability of nanoparticles is affected by their surface charge which also impacts their distribution in the bloodstream. Previous studies have shown that positively charged nanoparticles could be more effective in targeting tumour vessels. However, this has been replaced by neutrally charged nanoparticles which extravasate quicker into the tumour tissue [103].

## 11. Nanoparticles as a Treatment for Malignant Gliomas

A range of different nanoparticle formulations have been investigated to deliver chemotherapeutic drugs to the brain. The majority of these formulations have utilized polymers that have met the strict requirements needed to be accepted for biological applications [104]. During recent years, nanoparticles for the treatment of CNS diseases such as GBM have received significant attention [105,106]. With systemic administration of free drug, a small percentage of the drug crosses the BBB with non-specific accumulation in off target tissues resulting in serious unwanted side effects. Therefore, the use of nanoparticles for delivery to the brain has the potential to increase the percentage of drug that crosses the BBB while reducing non-specific accumulation in other tissues [107,108]. For example, gadolinium-loaded nanoparticles increased the level of gadolinium 100 fold when compared to free gadolinium [109]. The manufacture of nanoparticles has improved in the last few years with optimization of drug loading, encapsulation efficiency and release profile. Furthermore, improvements in the stealth capabilities of nanoparticles have increased their protection from agglutination with proteins in the blood, enabling them to avoid being cleared from the blood by the reticuloendothelial system (RES). Nanoparticles whose surface has been modified with ligands have been used to facilitate imaging of brain tumours nanoparticles [110,111]. PEGylation of nanoparticles has been widely used in drug delivery in order to protect nanoparticles from blood protein interaction and from the RES [112,113]. Dawson et al. demonstrated that the PEGylation of nanoparticles completely prevented their interaction with proteins in the plasma. However, other studies have shown that PEGlylation does not completely prevent the interaction between proteins and nanoparticles in the blood [114]. Nanoparticles offer a non-invasive method for drug delivery to the brain. However, they need to be optimized in relation to size, release kinetics, chemical properties, while modifying their surface could improve their ability to cross the BBB as well as protecting the drug from the biological environment and enhancing drug solubility [115]. Nanoparticles for drug delivery to the brain need to meet certain essential requirements to be most effective, with reduced toxicity. The requirements include non-toxic, biodegradable, prolonged circulation period, no aggregation in the blood, good encapsulation efficiency and the ability to cross the BBB [116].

## 12. Routes of Administration of Nanoparticles in the Treatment of Malignant Gliomas

There are three main routes of administration for nanoparticles designed to treat brain tumours: (1) direct delivery to the brain; (2) direct systemic delivery to the brain and (3) indirect systemic delivery to the brain. 

Direct delivery to the brain offers a way of bypassing the BBB by direct injection of the nanoparticles into the brain. CED has been used to infuse a nanoparticle suspension directly into brain tissue. Lollo et al. used CED to deliver 10 µL of paclitaxel-loaded lipid nanocapsules directly into the brain of mice. The results showed that the overall survival of mice treated with the lipid nanocapsules was significantly increased in comparison with mice treated with free paclitaxel [117,118]. Fourniols et al. described the direct injection of a photopolymerizable hydrogel containing TMZ-loaded micelles to the brain using a syringe to inject through an incision drilled in the skull. The TMZ-loaded micelles and injection were well tolerated while the hydrogel improved the drug release profile [119]. The major limitations of direct delivery to the brain is its invasive nature, the risk of infection and the need to control critical parameters such as pH and osmolarity which if not optimized may lead to brain damage [120]. Direct systemic delivery to the brain is where nanoparticles are directly administered into blood stream through carotid artery and transported to the brain avoiding the rest of the systemic circulation. This technique has shown improved survival compared to CED with reduced risk of brain damage [121]. Huynh et al. administered ferrociphekunol-loaded nanoparticles to the brain of GBM inflicted rats using both CED and direct systemic delivery. Direct systemic delivery provided a survival of 28 days compared to 24 days for the CED group. The results indicated that direct systemic delivery could provide a modest increase in survival when compared to direct delivery to the brain [120,122]. Indirect systemic delivery involves the delivery of nanoparticles into the systemic circulation via routes of administration that require absorption such as oral, topical, nasal, and peritoneal administration. The major advantages of oral administration are the convenience, non-invasiveness, and patient compliance. Kumar et al. administered two curcumin formulations (nanoparticles and plain suspension) orally to a rat intestinal ex-vivo model. The results showed that the bioavailability of nanoparticles formulation was 12 times greater than the plain suspension [123]. Intraperitoneal administration is widely used as an indirect systematic delivery method by injecting the drug into peritoneal tissue. It is used when administering large doses or when it is difficult to locate a vein for direct systemic delivery [124].

## 13. The Chemistry of Coating and Bioconjugation of Nanoparticles

Tumour tissues with their leaky vasculature allow the passive accumulation of particles from 10 to 200 nm in size by the EPR effect. However, chemotherapeutics penetration over time becomes challenging due to the increased interstitial pressure and the dense structure of the tumour tissues caused by hydrophobic regions in the brain extracellular space which block therapeutic particles, by steric hindrance, from accessing the tumour. Instead, chemotherapeutics diffuse towards the tumour edge and leak to blood vessels formed by angiogenesis. Additionally, nanoparticles are spotted by the RES as foreign particles and cleared by phagocytosis [125,126].

Advanced nanomedicine research has focused on developing stealth nanoparticles, which are designed with special coatings such as polyethylene glycol (PEG) that mask them from the RES system allowing them to circulate for longer in the bloodstream increasing tumour penetration and physiological stability. Furthermore, the coating enables targeting of brain tumour tissues in a more specific manner than uncoated particles [127,128]. Such coated nanoparticles are chemically and physically developed using different techniques which involve grafting, coprecipitation and surface adsorption [129]. Such bioconjugation involves using coatings that are either biological in nature or synthetic but specifically designed for biological applications [130].

Nanoparticles could have their surface modified through covalent and non-covalent ligand binding Covalent binding takes place via either disulphide bonds, primary amines cross linking, primary amine-carboxylic acid reaction, maleimide-thiol reaction, aldehyde-hydrazide reaction and primary amine-free aldehyde reaction [131]. Bioconjugates are also formed by using reactive crosslinking agents or reactive groups that aid in the coupling reaction. Some secondary activating agents could act as intermediates in the coupling process that facilitate binding specific functional groups. This process is specific in nature and resembles the selection of building blocks in order to create the whole structure. Affinity molecules act as an important part in the functionalized entity and they aim at targeting biomolecules [130].

Non-covalent or physical interaction between targeting ligands and nanoparticles does not involve chemical bonds, however, it may not create as strong a binding as is the case in covalent binding [132].

The process of PEGylation could take place by coupling with linear or branched PEG molecules which provides a more stable structure with enhanced water solubility and half-life while reducing cytotoxicity and adverse immune response [133,134].

Surfactants offer a major advantage for nanoparticles by helping them penetrate the BBB as well as enhancing their uptake by tumour cells. These surfactants could be polysaccharides, poloxamers or polysorbates. A study reported that Polysorbate 80 (p80) coated polymeric nanoparticles carrying paclitaxel had enhanced uptake by GBM cells due to the coating masking them from the P-gps, which are responsible for drug resistance, as well as by allowing their penetration of the BBB [135]. Previously published studies have demonstrated the beneficial role of p80 and Poloxamer 107 as surface ligands in facilitating transport across the BBB with proven efficacy in GBM rats [70,136,137,138]. However, the main issue with these particles is that they could only travel through endothelial cells to neurons in close proximity with the BBB, via cell to cell processes. As a result, the parenchyma barrier of the brain tumour will remain unpenetrated. Therefore, the focus on developing nanoparticles that can exceed this hurdle is necessary for a better treatment response. Another study used a similar approach of coating their PLGA nanoparticles with two different surfactants (p80 or poloxamer 188) for the intravenous delivery of two model drugs; loperamide and doxorubicin to the brain. Good efficacy was observed in rats containing an intracranial GBM when treated with doxorubicin loaded particles, while an analgesic effect was observed in mice when treated with the loperamide loaded particles, which confirms successful transport across the BBB at therapeutic levels. On the other hand, uncoated nanoparticles used in the same study had no effect for either drugs [127].

Other targeting moieties have been investigated for the treatment of malignant gliomas. Kuo and Chen (2015) reported that using lactoferrin and folic acid as grafting ligands for PLGA nanoparticles were effective in crossing the BBB and delivering etoposide in GBM U87MG cells [139]. Lactoferrin and folic acid coated nanoparticles had aided in the permeability of etoposide by almost 2-fold as compared to the uncoated nanoparticles. This resulted in a two-fold tumour suppression by the etoposide-loaded nanoparticles when compared to the free etoposide over 48 h. Furthermore, these nanoparticles were prepared with a cationic surfactant, didodecyl dimethyl ammonium bromide (DMAB) which was previously reported to enhance the affinity of the nanoparticles to the walls of arteries allowing for their uptake via AMT [140,141]. Other studies also highlighted the role of lactoferrin and other blood proteins (transferrin, insulin and leptin) as surface ligands in traversing through the BBB by RMT [77,139,142].

Another study demonstrated that a hydrogel made of polymeric micelles coated with polyethylene glycol dimethacrylate could provide sustained release of TMZ over a 1-week period in GBM bearing mice. This study indicated a dramatic decrease in the tumour volume following treatment with the TMZ photopolymerized hydrogel as well as increased apoptosis as compared with the other groups [119].

Dual coating of polymeric nanoparticles is also an interesting therapeutic trend. A study investigating polymeric nanoparticles with PEG and a covalently attached ligand called peptide-22 was shown to enhance BBB permeability, recognition by LDL receptor on the surface of glioma cells and increased delivery of paclitaxel [143]. The benefit of dual coating was further investigated in a study that coated PEGylated PCL nanoparticles with angiopep-2 which is a peptide that facilitates BBB permeation and drug delivery in glioma cells. This study has also employed cell penetrating peptides to allow deep permeation towards glioma cells and delivery of docetaxel which improved the rate of survival in glioma afflicted mice [144]. 

Another targeting protein moiety named EGFP-EGF1 was bound to polymeric nanoparticles carrying paclitaxel to form a dual coating alongside PEG aimed at targeting glioma in mice. This targeting protein has specific affinity towards tissue factor over-expressed in glioma cells therefore provided better penetration than non-fused nanoparticles. This study has reported enhanced apoptosis and necrosis and extended time of survival for mice treated with PLA-PEG-EGFP-EGF1 compared to the other groups [145].

An in vitro study using murine glioma cell lines C6 and F98 compared the cytotoxicity of etoposide loaded and unloaded PLGA nanoparticles with and without surface coatings. Their results have indicated enhanced cytotoxic effect on both glioma cell lines as compared to unloaded nanoparticles or free drug [146]. Surface coating of PLGA nanoparticles with protamine, which is a cationic protein that enhances drug transport across BBB, also significantly improved the delivery of cisplatin in bovine endothelial cells and also were cytotoxic in U87 GBM cells [147].

## 14. Drug-Loading of Nanoparticles

Drugs employed in the treatment of cancer can be loaded either by entrapment within, adsorption on or by covalently bonding to the nanoparticle [148]. The process of drug entrapment could take place either during or after the process of nanoparticles manufacture. Several factors, such as the solubility of the chemotherapy drug in the nanoparticles matrix, the molecular mass of the drug, the type of interaction between the drug and the nanoparticles and the functional groups on the surface of the nanoparticles influence their loading process [148]. 

The method of drug loading could result in drug within the core or on the surface of the nanoparticles. Drugs loaded on the surface by covalent bonding or physical adsorption as for example by electrostatic interactions between the nanoparticle and drug usually exhibit low stability and become pH liable [149,150]. Drugs entrapped within the nanoparticles usually have greater stability and tend be released over a sustained period of time.

## 15. Polymeric Nanoparticles for the Treatment of Malignant Gliomas

Polymeric nanoparticles are defined as submicron colloidal nanoparticles and are used as carriers for different drugs such as chemotherapeutic drugs which are either adsorbed on the surface or encapsulated within the nanoparticles [151]. There are many types of polymers which have been used in the manufacture of nanoparticles, such as poly(lactic acid) (PLA), poly(ε-caprolactone) (PCL), poly(butyl-cyanoacrylate) (PBCA), poly(glycolic acid) (PGA), PLGA and poly (amino acids). PLGA, PGA, PLA are the most extensively utilized polymers in drug delivery to the brain, because of their biocompatibility and low toxicity compared to other polymers [152]. They all degrade into lactic acid and glycolic acid that enter into the Krebs cycle where their metabolites are eliminated as carbon dioxide and water from the body [153]. Polymeric nanoparticles have advantages over other types of nanoparticles such as improved release kinetics, better compatibility with some active agents, no oxidation issues as with phospholipids and improved shelf-life [154,155,156]. The successful development of polymeric nanoparticles for drug delivery to the brain will require an understanding of the molecular weight, crystallinity and stability of the polymers as well as physicochemical properties of the drug [157].

The first polymeric nanoparticles developed to deliver drugs to the brain was performed by Kreuter et al. The BBB penetration of dalargin was significantly increased by formulating it into PBCA nanoparticles [158]. In 2001, Kreuter et al. used the same dalargin-loaded PBCA nanoparticles coated with p80, to increase penetration into brain tissue. This nanoparticle formulation was utilized for the delivery of other drugs, such as doxorubicin and loperamide into the brain [70]. Calvo et al. prepared PEG-PHDCA (poly(hexadecyl cyanoacrylate)) nanoparticles which demonstrated a greater accumulation in the brain when compared to the p80 formulation, which may be due to passive diffusion or intake via macrophage [159]. The density of the PEG coating on the surface of the nanoparticles can affect the level at which they cross the BBB. Vila et al. produced PEG-PLA nanoparicles with different densities of PEG coating and demonstrated that the smaller nanoparticles with the highest density of PEG had a greater accumulation in the brain [160].

## 16. Targeted Polymeric Nanoparticles for the Treatment of Malignant Gliomas

The active targeting of polymeric nanoparticles via surface modification with ligands that bind to target molecules on the surface of cancer cells or other cells within the body is a significant development in nanotechnology [161]. Table 1 shows examples of developed targeted polymer nanoparticles as targeted drug delivery system for treatment of malignant gliomas. The affinity ligands bind directly to antigens that are differentially overexpressed on the plasma membrane of cells or to extracellular proteins on the target tissue [161]. Active targeting of nanoparticles can be used for either extracellular or intracellular delivery of drugs. Nanoparticles are more effective if they are targeted to intracellular sites [154,162]. For example, Alexis et al. demonstrated increased cytotoxicty of paclitaxel-loaded nanoparticles using a ligand targeted to the extracellular domain of the trans-membrane human epidermal growth factor receptor 2 (HER-2) when compred with non trageted nanoparicles. This increase in cytotoxicty was due to an increased cellular uptake by the targeted nanoparticles [163]. Studies have confirmed that enhancing cellular uptake is the most important role of using targeted nanoparticles [164,165]. Targeted nanoparticles can have either single or multi ligands attached to their surfaces. To date, most researchers have prefered to use a single ligand as multi-ligands are associated with some disadvantges, particulalry when used for penetration of the BBB and tumour cells. For example, multi-ligands influence drug release as well as the mobility of the nanoparticles. Furthermore, competitive binding and/or an interaction between ligands may reduce the targeting effeciency of the nanoparticles [166].

Transferrin receptors and low density lipoprotein receptor related protein (LRP) are known to be overexpressed on glioma cells [166]. These two receptors have been used to target polymeric nanoparticles to glioma cells by attaching the anti-transferrin and angiopep ligands to their surface. Anti-transferrin can cross the BBB through transferrin receptors while the angiopep ligand binds to LRP on the surface of glioma cells [166,171]. The transferrin receptor is the most widely characterized receptor-mediated transport system, which provides an efficient cellular uptake and is over expressed in numerous tumour cells [167]. The targeting of BBB of an in vitro model has increased 20-fold with transferrin-PLGA nanoparticles compared to non-coated PLGA nanoparticles. Chang et al. demonstrated, using an in vitro model of the BBB, that transferrin-PLGA nanoparticles had a 20 fold increase in uptake by the BBB when compared to un-coated PLGA nanoparticles. The uptake of the transferrin-PLGA nanoparticles by the BBB was by endocytosis [167]. The major disadvantages of using transferrin as a ligand for nanoparticles is the competition with endogenous transferrin for receptor binding. This may result in reducing the cellular uptake and thus the effectiveness of the nanoparticles [178]. An antibody directed against the transferrin has been used as an alternative ligand to the endogenous transferrin as it binds to an epitope of the transferrin receptor which is located at a different location from transferrin binding. Therefore, the nanoparticles have less binding competition as they do not interfere with the transferrin intake mechanism. This will increase their cellular uptake and thus their effectivness [179]. Different antibodies such as OX26 (anti-rat TfR mAbs), R17-217 and 8D3 (both anti-mouse TfR mAbs) have been developed for enhancing brain uptake. OX26 mAbs has been shown to have a high affinity for cells that overexpress transferrin, including GBM cells [180,181]. The level of brain uptake for each of the antibodies is different. For example, the brain uptake of 8D3 mAbs was relatively high compared to R17-217 mAbs. 8D3 and R17-217 mAbs were more selective for brain than liver and kidney [182]. Rmalho et al. developed receptor mediated TMZ-loaded PLGA nanoparticles for GBM treatment functionalized with an OX26 mAbs. The cellular internalization of the OX26mAbs nanoparticles was signifcantly enhanced compared to the PLGA nanoparticles with no mAbs [176].

Another approach to enhancing the uptake of nanoparticles by the BBB is to coat them with a polymer that will facilitate cellular uptake. As discussed earlier about the role of surfactants in coating, Kreuter demonstrated that intravenously injected doxorubicin-loaded p80-coated nanoparticles had a 40% cure rate in rats with intracranially transplanted GBMs. Although not fully elucitated, he hypothesised that the most likely mechanism for transport of the nanoparticles across the BBB was endocytosis by the endothelial cells lining the brain blood capillaries. Coating the nanoparticles with p80 lead to the adsorption of apolipoprotein E from blood plasma onto the nanoparticles’ surface. The particles then mimiced LDL particles and could thus interact with the LDL receptor leading to their enhanced uptake by the endothelial cells [70]. The first polymeric nanoparticles for penetration of the BBB were investigated by Schröder et al. in 1995. PBCA nanoparticle coated with p80 enhanced BBB penetration of hexapeptide dalargin-loaded nanoparticles [183]. Wohlfart et al. demonstrated, using a rat glioma model, that poloxamer 188-coated PLGA nanoparticles enabled the delivery of doxorubicin across the BBB in the therapeutically effective concentrations. The basis for their transport across the BBB was hypothesised to be adsorption of blood apolipoproteins (ApoE or ApoA-I) onto the nanoparticles surface due to the poloxamer 188 coating, followed by RMT of the nanoparticles [184]. Manlioovskaya et al. demonstrated that these same nanoparticles entered U87 human GBM cells via clathrin-mediated endocytosis. They also demonstrated that the nanoparticles released their doxorubicin via diffusion rather than by intracellular degradation [175]. These studies prove that PLGA nanoparticles coated with poloxamer 188 could improve the delivery of doxorubicin and potentially other chemotherapeutic drugs into brain tumours.

Another promising LPR ligand for delivering nanoparticles to the CNS and BBB penetration is angiopep. It is from a peptide family that is derived from aprotinin and human proteins [185]. The transcytosis capacity and parenchymal accumulation of angiopep-2 is much greater compared to transferrin [186]. The ability of angiopep to facilitate penetration of the BBB of polymeric nanoparticles has been confirmed in a number of studies [169,187]. Xin et al. fabricated dual targeting nanoparticles to improve the drug delivery of paclitaxel to glioma cells. Angiopep-PEG-PCL nanoparticles were highly endocytosed by U87 GBM cells compared with non-targeted PEG-PCL nanoparticles. These nanoparticles have also shown higher penetration, distribution, and accumulation in 3D glioma spheroids as well as increased efficacy in U87 tumour bearing mice [169,171].

## 17. Challenges Associated with Nanomedicine as a Treatment for Malignant Gliomas

### 17.1. Reticuloendothelial System

The RES also termed the mononuclear phagocyte system (MPS), possesses cellular and noncellular components. Phagocytes could cause the clearance of nanoparticles by binding to them and triggering cytokine cascade, which causes inflammation [188]. Moreover, macromolecules such as proteins and lipids and others could attach to the surface of the nanoparticles forming a biological corona that gets recognized by the immune system and cleared from the blood stream [189]. This challenge could be overcome by surface modification of the nanoparticles that could conceal them from being recognized by the RES and allow their existence for longer periods in bloodstream. Surface modification is done using zwitterionic ligands such as cysteine, glutathione or by PEGylation [188]. In a study performed by Choi et al. using zwitterionic (cysteine) or neutral dihydrolipoic acid ((DHLA)-connected polyethylene glycol; DHLA-PEG) coatings to coat quantum dots has prevented adsorption of serum proteins and enhanced their renal clearance [190]. An in vivo study showed that using nanoparticles of PEGylated human serum albumin loaded with paclitaxel accomplished long systemic circulation of more than 96 h and enhanced accumulation in the tumour providing high efficacy against cancer and extension in the life span of animals [191]

### 17.2. Renal System

The main obstacle facing nanoparticles with the renal system is the process of blood filtration. Nanoparticles will follow certain routes by passing through fenestrated endothelium that has 70–100 nm pores, then they will go through the capillary endothelium and podocytes that are programmed to clear particles sized between 2 and 8 nm, whereas nanoparticles >8 nm will face difficulty crossing the glomerular filtration barrier. In addition, the fact that the glomerular basement membrane carries a negative charge, cationic nanoparticles (2–6 nm) will exhibit more renal clearance than neutral or anionic same sized nanoparticles [188]. The shape of a nanoparticle could also influence renal clearance, with enhanced clearance of rod shaped nanoparticles of size 0.8–1.2 nm diameters as reported by Ruggiero et al. [192]. Size exclusion is a major challenge that affects the overall benefit of using the nanoparticles. The solution to this problem could lie through developing nanoparticles of biodegradable materials that can break down into particles prone to renal clearance. However, this could result in premature release of the therapeutic agents before reaching their target site [193]. Therefore, in designing nanomedicines for clinical applications, it is necessary to keep the balance between formulating nanoparticles that have renal clearance to avoid long term toxicity as well as maintaining the therapeutic levels of the drugs in the plasma [188]. 

### 17.3. Blood Brain Barrier

As mentioned previously, the BBB is a barrier consisting of tight junctions that limit the entry of nanoparticles into the brain. However, nanoparticles with ligands attached have been used to pass through the BBB by the receptor mediated endocytosis [188].

### 17.4. Pathophysiological Barriers in Cancer

The composition and structure of the tumour extracellular matrix and its vasculature vary according to the nature of the cancer, it’s position and stage, alongside personal characteristics [188]. Therefore, deep penetration of the nanoparticles could be difficult to achieve [194]. Three steps are involved in the transport of pharmaceutical agents to tumour cells. These involve flow of the nanoparticles through blood vessels, then passage through the walls of the blood vessels, eventually crossing the interstitial space to reach the tumour site. The morphological discrepancy between tumour and normal tissues affect the delivery of the therapeutic agents. The abnormal environment of tumourous tissues results in leaky vessels, abnormal blood flow, dysfunctional lymphatic vessels and vascular hyperpermeability that causes interstitial hypertension. The high pressure of the interstitial fluid and dense extracellular matrix hinder the process of diffusion [195,196]. Several strategies have been addressed to enhance drug delivery such as: (1) Normalization of tumour vasculature by using antiangiogenic agents that repair the imbalance that took place between the overexpressed proangiogenic and antiangiogenic factors in tumour tissues [196]. This approach has rapidly normalized the tumour microenvironment and reduced vessel size and vascular permeability in GBM patients after receiving cediranib as an antiangiogenic therapy [197]. In a preclinical study, surface modified nanoparticles (20–40 nm) could successfully penetrate into breast cancer tissues following vascular normalization therapy [198]. Nevertheless, normalized vasculature would not grant entry to extra large nanoparticles due to the reduced pore vessel size. In addition, vessel normalization is impermanent and requires drug administration within the normalization period [196]. (2) Normalization of tumour matrix that is mainly composed of collagen and glycosaminoglycan. The normalization process is based on degrading such components to improve nanoparticles penetration. Bacterial collagenase treatment has been administered in high collagen containing tumours such as HSTS26T sarcoma and Mu89 melanoma and has improved penetration of IgG antibodies (4.5 nm size) by two fold [199]. Also, interstitial distribution of herpes simplex virus (size 75 nm) was improved by three-fold [200]. Other strategies have been suggested to improve penetration of nanoparticles into cancerous tissues. Inhibiting growth factor-β in pancreatic adenocarcinoma was reported to enhance the penetration of polymeric nanoparticles 100 nm in size [201]. Alternatively, using multi-staged nanoparticles could enhance drug delivery in cancer. This involves using large nanoparticles that have longer half-life in the blood stream [202]. These large particles dissociate upon entry into the tumour microenvironment and release smaller nanoparticles that diffuse on a deeper level into the tumour tissue. A multistage nanoparticle system (100 nm) was engineered with a gelatin core to dissociate and release nanoparticles (10 nm) when it comes in contact with matrix metalloproteinases, for a deeper tumour penetration [203].

Designing nanoparticles that can have deep penetration into tumour tissues are under development. Additionally, smart nanoparticles are being developed that can respond to the surrounding conditions and allow a better bioavailability for treatment [204].

### 17.5. Multidrug Resistance

Multidrug resistance (MDR), whether hereditary or gained by long term exposure to drugs, involves discharge of drugs outside the cells leading to reduced drug concentration and efficacy inside the cell lumen. Cancer cells can be resistant to chemotherapeutic drugs causing increased toxicity of healthy cells which get exposed to drugs that get ejected by cancer cells. Some chemotherapy drugs that cancer cells are resistant to include taxanes, anthracyclines and vinca alkaloids [205]. In cancer, MDR usually comes from overexpressed P-gp which is an ATP-binding cassette (ABC) transporter that acts as an efflux pump with the ability of binding many various hydrophobic drugs [206]. Such transporter is present in several organs such as brain, liver and placenta, for example and it functions by protecting organs from toxins [207]. Some other MDR associated proteins involve MDR-associated protein-1 and the breast cancer resistance protein (BCRP) [208]. Efflux pump inhibitors such as verapamil (covera) and cyclosporine have been investigated and are emerging as first-generation antagonists [209]. Addressing MDR in cancer has involved the exploitation of nanoparticles drug delivery systems in encapsulating chemotherapy drugs. Liposomes nanoparticles encapsulating doxorubicin and verapamil have been formulated for the targeted inhibition of P-gp [210]. Furthermore, hybrid nanoparticles of lipids and co-polymers were developed and loaded with doxorubicin and GG918 to target BCRP [211]. Both of the previously stated studies have accomplished higher cytotoxicity against leukemia and breast cancer cell lines, respectively, compared to free drug administration. PCL polymer was also employed alongside other co-polymers in the development of micelle nanoparticles of siRNA to target MDR-1 and perform silencing of the gene responsible for P-gp expression [212].

## 18. Clinical Transition of Polymeric Nanoparticles

Polymeric nanoparticle formulations utilizing different polymers, coatings and targeting ligands have been introduced into the clinic. As can be seen from above a wide range of different nanoparticle delivery systems have been investigated pre-clinically, which may lead to more nanoparticles reaching the clinic. Furthermore the FDA has approved a range of different routes of administration for nanoparticles, such as systemic, local, and oral administration [213]. The main route of administration used in most preclinical and clinical studies is intravenous administration due to the nanoparticles being able to reach all parts of the body giving them a high potential to influence clinical care by targeting both the primary cancer and any associated metastasis [214,215]. All of the nanoparticle formulations approved for cancer treatment are liposomal formulations. The first approved nanoparticle formulation was PEG-functionalized liposomal doxorubicin (Doxil) in 1995, and the most recently approved nanoparticle is irinotecan liposomal formulation (Onivyde) [216,217]. The majority of all approved liposomal nanoparticle formulations are not PEGylated except for Doxil and Onivyde, which have been shown to have advantages over non-PEGylated nanoparticle formulations even with their low amount of PEG [213,218]. Preclinical research performed in the 1970s, 1980s, and 1990s, using polymers as controlled release systems for drug delivery has led to a number of clinically approved products [161]. The clinical success of using polymers for controlled release, and the ability to manufacture polymeric formulations on the nanoscale have driven the research of polymeric depots away from the macro/micro scale to the nano scale [219]. A Number of PEGylated polymeric nanoparticle formulations such as SP1049C, NK911, and Genexol-PM are in early phase clinical trials for various types of cancers [220,221,222,223,224]. SP1049C is a pluronic polymeric micelle nanoparticle containg entrapped doxorubicin, that is currently evaluated for patients with esophagus and esophageal junction metastatic cancer in a phase II clinical trial [220]. The other two polymeric nanoparticles, K911 (doxorubicin-loaded PEG-poly(aspartic acid)) and Genexol-PM (paclitaxel-loaded PEG-PLA) are in phase II clinical trials for various cancers [221,223]. These first generation, polymeric nanoparticles have shown promising effect for various cancers with wider therapeutic windows and lower side effects. However, these nanoparticles are associated with number of limitations related to targeting, therefore targeted polymeric nanoparticles are now under preclinical and clinical investigation [161]. The first targeted polymeric nanoparticles to reach the clinic is BIND-014, which is composed of a prostate specific membrane antigen (PSMA) conjugated to a docetaxel-loaded PLGA nanoparticles [161,223]. There are only two other targeted polymeric nanoparticles currently in clinical trials, CALAA-01 (phase I), and SEL-068 (phase I) [225,226,227]. The synthesis of targeted nanoparticles is complex and is difficult to scale-up. The tuning of ligand density is very difficult because the target ligands attach to the surface of the pre-prepared nanoparticles through post-coupling processes. In order to achieve a high efficiency of the coupling of the ligands excessive amounts of reagents and purification techniques for removing unbound ligands are needed. These issues have lead to batch-to-batch variability and difficulty in reproducing the surface properties. Consequently, the clinical transition for targeted nanoparticles will be difficult unless they are prepared using pre-functionalized polymeric materials and minimum number of components [161,228]. However, this will require the development and registration of new polymeric excipients.

## 19. Conclusions

Malignant gliomas remain to be among the most aggressive forms of tumours that may not respond to most of the conventional treatments of chemotherapy and RT. This in fact is attributed to the selective nature of the BBB that prevents most particles from entry inside the brain including therapeutics. Moreover, conventional management strategies for glioma only allow patients some extra time to survive while struggling with deleterious side effects that develop mostly from the invasiveness of the treatment approaches. Nanomedicine is a flexible and non-invasive therapeutic field that allows the design of materials with nanometer size dimensions to act as drug carriers and delivery agents crossing the BBB via targeting ligands and special coatings. Such designed nanoparticles will aim only towards receptors of interest that are overexpressed on tumour tissues, for instance, while sparing normal tissues. The promising pre-clinical data have paved the way for more nanoparticles to be introduced in the clinic. The FDA has approved various routes of administration of nanoparticles with a preference towards the intravenous route which offers advantages towards treatment of metastasized tumours. Polymeric nanoparticles are gaining more attention for the treatment of malignant gliomas owing to their biodegradable and biocompatible behaviour inside the human body and the unlimited designs and characteristics they can be manipulated into. Polymeric nanoparticles can be extra advantageous when PEGylated as discussed earlier in this review. However, further efforts are needed to optimize their size, drug loading capacity and release of hydrophilic and hydrophobic drugs, taking in consideration the different physicochemical properties of drugs and the physiological barriers that may hamper their success.

## Figures and Tables

**Table 1:** Examples of developed targeted polymer nanoparticles as targeted drug delivery system for treatment of brain tumours malignant gliomas.

Polymer Type	Loaded	Size (nm)	Targeting Strategy	Targeted Site	Ref.
PLGA	Dil	90	Transferrin	Transferrin receptors	[167]
PMLA	Antisense ON	25	mAbs antisense oligonucleotides (AONs)	(proteins) laminin-411	[168]
PEG-PCL	Paclitaxel	<100	Angiopep	LRP	[169]
PEG-PLGA	Coumarin 6	125	Peptide (12 amino-acid)	Peptide	[170]
PEG-PCL	Paclitaxel Rhodamine	90	Angiopep	LRP	[171]
PLGA	Methotrexate	85	Transferrin	Transferrin receptors	[166]
PEG-PLGA	Doxorubicin	100–300	Endogenous tripeptide thiol (glutathione)	Glutathione transporters	[172]
PLGA	Loperamide	100	mAbs (8D3)	Transferrin receptors	[173]
PLGA	Curcumin	100	Magnetic guidance Peptide (T7)	Transferrin receptors	[174]
PLGA	Doxorubicin	120	Poloxamer 188	LRP	[175]
PEG-PLGA	TMZ	19	mAbs (OX26)	Transferrin receptors	[176]
PLGA	Paclitaxel	230–255	Tripeptide(RGD) Superparamac iron oxide (SPIO)	αvβ3 integrin	[177]

## References

[1] Kaufmann J.K., Chiocca E.A. (2014). Glioma virus therapies between bench and bedside. Neuro-Oncology.

[2] Silva C.O., Pinho J.O., Lopes J.M., Almeida A.J., Gaspar M.M., Reis C. (2019). Current trends in cancer nanotheranostics: Metallic, polymeric, and lipid-based systems. Pharmaceutics.

[3] Lapointe S., Perry A., Butowski N.A. (2018). Primary brain tumours in adults. Lancet.

[4] Paw I., Carpenter R.C., Watabe K., Debinski W., Lo H.-W. (2015). Mechanisms regulating glioma invasion. Cancer Lett..

[5] Cornago M., Garcia-Alberich C., Blasco-Angulo N., Vall-Llaura N., Nager M., Herreros J., Comella J., Sanchis D., Llovera M. (2014). Histone deacetylase inhibitors promote glioma cell death by G2 checkpoint abrogation leading to mitotic catastrophe. Cell Death Dis..

[6] De Boer A.G., Gaillard P.J. (2007). Strategies to improve drug delivery across the blood-brain barrier. Clin. Pharmacokinet..

[7] Parrish K.E., Cen L., Murray J., Calligaris D., Kizilbash S., Mittapalli R.K., Carlson B.L., Schroeder M.A., Sludden J., Boddy A.V. (2015). Efficacy of PARP inhibitor rucaparib in orthotopic glioblastoma xenografts is limited by ineffective drug penetration into the central nervous system. Mol. Cancer Ther..

[8] Van Tellingen O., Yetkin-Arik B., De Gooijer M., Wesseling P., Wurdinger T., De Vries H. (2015). Overcoming the blood–brain tumor barrier for effective glioblastoma treatment. Drug Resist. Updates.

[9] Binello E., Germano I.M. (2011). Targeting glioma stem cells: A novel framework for brain tumors. Cancer Sci..

[10] Dolecek T.A., Propp J.M., Stroup N.E., Kruchko C. (2012). CBTRUS statistical report: Primary brain and central nervous system tumors diagnosed in the United States in 2005–2009. Neuro-Oncology.

[11] MacKenzie D. (1926). A classification of the tumours of the glioma group on a histogenetic basis with a correlated study of prognosis. Can. Med. Assoc. J..

[12] Louis D.N., Perry A., Reifenberger G., Von Deimling A., Figarella-Branger D., Cavenee W.K., Ohgaki H., Wiestler O.D., Kleihues P., Ellison D.W. (2016). The 2016 World Health Organization classification of tumors of the central nervous system: A summary. Acta Neuropathol..

[13] Sathornsumetee S., Rich J.N., Reardon D.A. (2007). Diagnosis and treatment of high-grade astrocytoma. Neurol. Clin..

[14] Fischer U., Meese E. (2007). Glioblastoma multiforme: The role of DSB repair between genotype and phenotype. Oncogene.

[15] Hess K.R. (1999). Extent of resection as a prognostic variable in the treatment of gliomas. J. Neuro-Oncol..

[16] Simpson J., Horton J., Scott C., Curran W., Rubin P., Fischbach J., Isaacson S., Rotman M., Asbell S., Nelson J. (1993). Influence of location and extent of surgical resection on survival of patients with glioblastoma multiforme: Results of three consecutive Radiation Therapy Oncology Group (RTOG) clinical trials. Int. J. Radiat. Oncol. Biol. Phys..

[17] Fadul C., Wood J., Thaler H., Galicich J., Patterson R., Posner J. (1988). Morbidity and mortality of craniotomy for excision of supratentorial gliomas. Neurology.

[18] Hentschel S.J., Sawaya R. (2003). Optimizing outcomes with maximal surgical resection of malignant gliomas. Cancer Control.

[19] Iacob G., Dinca E.B. (2009). Current data and strategy in glioblastoma multiforme. J. Med. Life.

[20] Van den Bent M., Afra D., De Witte O., Hassel M.B., Schraub S., Hoang-Xuan K., Malmström P., Collette L., Piérart M., Mirimanoff R. (2005). Long-term efficacy of early versus delayed radiotherapy for low-grade astrocytoma and oligodendroglioma in adults: The EORTC 22845 randomised trial. Lancet.

[21] Chandana S.R., Movva S., Arora M., Singh T. (2008). Primary brain tumors in adults. Am. Fam. Physician.

[22] Walker R.A. (1978). Significance of alpha-subunit HCG demonstrated in breast carcinomas by the immunoperoxidase technique. J. Clin. Pathol..

[23] Shapiro W.R., Green S.B., Burger P.C., Mahaley M.S., Selker R.G., VanGilder J.C., Robertson J.T., Ransohoff J., Mealey J., Strike T.A. (1989). Randomized trial of three chemotherapy regimens and two radiotherapy regimens in postoperative treatment of malignant glioma: Brain Tumor Cooperative Group Trial 8001. J. Neurosurg..

[24] Heesters M., Wijrdeman H., Struikmans H., Witkamp T., Moerland M. (1993). Brain tumor delineation based on CT and MR imaging. Implications for radiotherapy treatment planning. Strahlentherapie und Onkologie.

[25] Saran R., Robinson B., Abbott K.C., Agodoa L.Y., Bhave N., Bragg-Gresham J., Balkrishnan R., Dietrich X., Eckard A., Eggers P.W. (2018). US renal data system 2017 annual data report: Epidemiology of kidney disease in the United States. Am. J. Kidney Dis. Off. J. Natl. Kidney Found..

[26] Scaringi C., Agolli L., Minniti G. (2018). Technical Advances in Radiation Therapy for Brain Tumors. Anticancer Res..

[27] Stupp R., Mason W.P., Van Den Bent M.J., Weller M., Fisher B., Taphoorn M.J., Belanger K., Brandes A.A., Marosi C., Bogdahn U. (2005). Radiotherapy plus concomitant and adjuvant temozolomide for glioblastoma. N. Engl. J. Med..

[28] Stupp R., Roila F., Group E.G.W. (2009). Malignant glioma: ESMO clinical recommendations for diagnosis, treatment and follow-up. Ann. Oncol..

[29] De Vleeschouwer S., Bergers G. (2017). Glioblastoma: To target the tumor cell or the microenvironment. Glioblastoma [Internet].

[30] Stupp R., Hegi M.E., Van Den Bent M.J., Mason W.P., Weller M., Mirimanoff R.O., Cairncross J.G., European Organisation for Research and Treatment of Cancer, Brain Tumour and Radiotherapy Groups’, National Cancer Institute of Canada Clinical Trials Group (2006). Changing paradigms—An update on the multidisciplinary management of malignant glioma. Oncologist.

[31] Stupp R., Taillibert S., Kanner A.A., Kesari S., Steinberg D.M., Toms S.A., Taylor L.P., Lieberman F., Silvani A., Fink K.L. (2015). Maintenance therapy with tumor-treating fields plus temozolomide vs temozolomide alone for glioblastoma: A randomized clinical trial. JAMA.

[32] Stupp R., Wong E.T., Kanner A.A., Steinberg D., Engelhard H., Heidecke V., Kirson E.D., Taillibert S., Liebermann F., Dbalý V. (2012). NovoTTF-100A versus physician’s choice chemotherapy in recurrent glioblastoma: A randomised phase III trial of a novel treatment modality. Eur. J. Cancer.

[33] Davis M.E. (2013). Tumor treating fields-an emerging cancer treatment modality. Clin. J. Oncol. Nurs..

[34] Barani I.J., Larson D.A. (2015). Radiation therapy of glioblastoma. Current Understanding and Treatment of Gliomas.

[35] Norden A.D., Wen P.Y. (2006). Glioma therapy in adults. Neurologist.

[36] Kostaras X., Cusano F., Kline G., Roa W., Easaw J. (2014). Use of dexamethasone in patients with high-grade glioma: A clinical practice guideline. Curr. Oncol..

[37] Wen P.Y., Schiff D., Kesari S., Drappatz J., Gigas D.C., Doherty L. (2006). Medical management of patients with brain tumors. J. Neuro-Oncol..

[38] Omuro A., DeAngelis L.M. (2013). Glioblastoma and other malignant gliomas: A clinical review. JAMA.

[39] Thakkar J.P., Dolecek T.A., Horbinski C., Ostrom Q.T., Lightner D.D., Barnholtz-Sloan J.S., Villano J.L. (2014). Epidemiologic and molecular prognostic review of glioblastoma. Cancer Epidemiol. Prev. Biomark..

[40] Wick W., Hartmann C., Engel C., Stoffels M., Felsberg J., Stockhammer F., Sabel M.C., Koeppen S., Ketter R., Meyermann R. (2009). NOA-04 randomized phase III trial of sequential radiochemotherapy of anaplastic glioma with procarbazine, lomustine, and vincristine or temozolomide. J. Clin. Oncol..

[41] Cairncross G., Wang M., Shaw E., Jenkins R., Brachman D., Buckner J., Fink K., Souhami L., Laperriere N., Curran W. (2013). Phase III trial of chemoradiotherapy for anaplastic oligodendroglioma: Long-term results of RTOG 9402. J. Clin. Oncol..

[42] Ellor S.V., Pagano-Young T.A., Avgeropoulos N.G. (2014). Glioblastoma: Background, Standard Treatment Paradigms, and Supportive Care Considerations.

[43] Walid M.S. (2008). Prognostic factors for long-term survival after glioblastoma. Perm. J..

[44] Ostrom Q.T., Gittleman H., Liao P., Rouse C., Chen Y., Dowling J., Wolinsky Y., Kruchko C., Barnholtz-Sloan J. (2014). CBTRUS statistical report: Primary brain and central nervous system tumors diagnosed in the United States in 2007–2011. Neuro-Oncology.

[45] Ostrom Q.T., Gittleman H., Fulop J., Liu M., Blanda R., Kromer C., Wolinsky Y., Kruchko C., Barnholtz-Sloan J.S. (2015). CBTRUS statistical report: Primary brain and central nervous system tumors diagnosed in the United States in 2008–2012. Neuro-Oncology.

[46] Lovely M.P., Stewart-Amidei C., Arzbaecher J., Bell S., Maher M.E., Maida M., Mogensen K., Nicolaseau G. (2014). Care of the adult patient with a brain tumor. J. Neurosci. Nurs..

[47] Tomasetti C., Li L., Vogelstein B. (2017). Stem cell divisions, somatic mutations, cancer etiology, and cancer prevention. Science.

[48] Wilson T.A., Karajannis M.A., Harter D.H. (2014). Glioblastoma multiforme: State of the art and future therapeutics. Surg. Neurol. Int..

[49] Verhaak R.G., Hoadley K.A., Purdom E., Wang V., Qi Y., Wilkerson M.D., Miller C.R., Ding L., Golub T., Mesirov J.P. (2010). Integrated genomic analysis identifies clinically relevant subtypes of glioblastoma characterized by abnormalities in PDGFRA, IDH1, EGFR, and NF1. Cancer Cell.

[50] Phillips H.S., Kharbanda S., Chen R., Forrest W.F., Soriano R.H., Wu T.D., Misra A., Nigro J.M., Colman H., Soroceanu L. (2006). Molecular subclasses of high-grade glioma predict prognosis, delineate a pattern of disease progression, and resemble stages in neurogenesis. Cancer Cell.

[51] Hart M.G., Garside R., Rogers G., Stein K., Grant R. (2013). Temozolomide for high grade glioma. Cochrane Database Syst. Rev..

[52] Ashby L.S., Smith K.A., Stea B. (2016). Gliadel wafer implantation combined with standard radiotherapy and concurrent followed by adjuvant temozolomide for treatment of newly diagnosed high-grade glioma: A systematic literature review. World J. Surg. Oncol..

[53] Brem H., Ewend M.G., Piantadosi S., Greenhoot J., Burger P.C., Sisti M. (1995). The safety of interstitial chemotherapy with BCNU-loaded polymer followed by radiation therapy in the treatment of newly diagnosed malignant gliomas: Phase I trial. J. Neuro-Oncol..

[54] Fung L.K., Ewend M.G., Sills A., Sipos E.P., Thompson R., Watts M., Colvin O.M., Brem H., Saltzman W.M. (1998). Pharmacokinetics of interstitial delivery of carmustine, 4-hydroperoxycyclophosphamide, and paclitaxel from a biodegradable polymer implant in the monkey brain. Cancer Res..

[55] Friedman H.S., Kerby T., Calvert H. (2000). Temozolomide and treatment of malignant glioma. Clin. Cancer Res..

[56] Chang J.E., Khuntia D., Robins H.I., Mehta M.P. (2007). Radiotherapy and radiosensitizers in the treatment of glioblastoma multiforme. Clin. Adv. Hematol Oncol..

[57] Scott J., Tsai Y.-Y., Chinnaiyan P., Yu H.-H.M. (2011). Effectiveness of radiotherapy for elderly patients with glioblastoma. Int. J. Radiat. Oncol. Biol. Phys..

[58] Petty M.A., Lo E.H. (2002). Junctional complexes of the blood–brain barrier: Permeability changes in neuroinflammation. Prog. Neurobiol..

[59] De Boer A., Breimer D. (1998). Cytokines and blood-brain barrier permeability. Progress in Brain Research.

[60] Wahl M., Unterberg A., Baethmann A., Schilling L. (1988). Mediators of blood-brain barrier dysfunction and formation of vasogenic brain edema. J. Cereb. Blood Flow Metab..

[61] Kastin A.J., Pan W., Maness L.M., Banks W.A. (1999). Peptides crossing the blood–brain barrier: Some unusual observations. Brain Res..

[62] M Rabanel J., Aoun V., Elkin I., Mokhtar M., Hildgen P. (2012). Drug-loaded nanocarriers: Passive targeting and crossing of biological barriers. Curr. Med. Chem..

[63] Habgood M., Begley D., Abbott N. (2000). Determinants of passive drug entry into the central nervous system. Cell. Mol. Neurobiol..

[64] Hawkins B.T., Davis T.P. (2005). The blood-brain barrier/neurovascular unit in health and disease. Pharmacol. Rev..

[65] Jain K.K. (2012). Nanobiotechnology-based strategies for crossing the blood–brain barrier. Nanomedicine.

[66] Shityakov S., Förster C. (2014). In silico predictive model to determine vector-mediated transport properties for the blood–brain barrier choline transporter. Adv. Appl. Bioinform. Chem. AABC.

[67] Yu A.S., Hirayama B.A., Timbol G., Liu J., Diez-Sampedro A., Kepe V., Satyamurthy N., Huang S.-C., Wright E.M., Barrio J.R. (2012). Regional distribution of SGLT activity in rat brain in vivo. Am. J. Physiol.-Cell Physiol..

[68] Miller D.S. (2015). Regulation of ABC transporters blood–brain barrier: The good, the bad, and the ugly. Advances in Cancer Research.

[69] On N.H., Miller D.W. (2014). Transporter-based delivery of anticancer drugs to the brain: Improving brain penetration by minimizing drug efflux at the blood-brain barrier. Curr. Pharm. Des..

[70] Kreuter J. (2001). Nanoparticulate systems for brain delivery of drugs. Adv. Drug Deliv. Rev..

[71] Bray D., Levin M.D., Morton-Firth C.J. (1998). Receptor clustering as a cellular mechanism to control sensitivity. Nature.

[72] Ehrlich M., Boll W., Van Oijen A., Hariharan R., Chandran K., Nibert M.L., Kirchhausen T. (2004). Endocytosis by random initiation and stabilization of clathrin-coated pits. Cell.

[73] Giddings S., Chirgwin J., Permutt M. (1985). Evaluation of rat insulin messenger RNA in pancreatic and extrapancreatic tissues. Diabetologia.

[74] Skarlatos S., Yoshikawa T., Pardridge W.M. (1995). Transport of [125I] transferrin through the rat blood-brain barrier. Brain Res..

[75] Zhang Y., Pardridge W.M. (2001). Rapid transferrin efflux from brain to blood across the blood–brain barrier. J. Neurochem..

[76] Schlachetzki F., Zhu C., Pardridge W.M. (2002). Expression of the neonatal Fc receptor (FcRn) at the blood–brain barrier. J. Neurochem..

[77] Golden P.L., Maccagnan T.J., Pardridge W.M. (1997). Human blood-brain barrier leptin receptor. Binding and endocytosis in isolated human brain microvessels. J. Clin. Investig..

[78] Mellman I. (1996). Endocytosis and molecular sorting. Annu. Rev. Cell Dev. Biol..

[79] Wang Z.H., Wang Z.Y., Sun C.S., Wang C.Y., Jiang T.Y., Wang S.L. (2010). Trimethylated chitosan-conjugated PLGA nanoparticles for the delivery of drugs to the brain. Biomaterials.

[80] Hervé F., Ghinea N., Scherrmann J.-M. (2008). CNS delivery via adsorptive transcytosis. AAPS J..

[81] Batrakova E.V., Gendelman H.E., Kabanov A.V. (2011). Cell-mediated drug delivery. Expert Opin. Drug Deliv..

[82] Masserini M. (2013). Nanoparticles for brain drug delivery. ISRN Biochem..

[83] Jain S., Mishra V., Singh P., Dubey P., Saraf D., Vyas S. (2003). RGD-anchored magnetic liposomes for monocytes/neutrophils-mediated brain targeting. Int. J. Pharm..

[84] Qin J., Chen D., Hu H., Cui Q., Qiao M., Chen B. (2007). Surface modification of RGD-liposomes for selective drug delivery to monocytes/neutrophils in brain. Chem. Pharm. Bull..

[85] Rodrigueza W.V., Pritchard P.H., Hope M.J. (1993). The influence of size and composition on the cholesterol mobilizing properties of liposomes in vivo. Biochim. Biophys. Acta (BBA)-Biomembr..

[86] Bennewitz M.F., Saltzman W.M. (2009). Nanotechnology for delivery of drugs to the brain for epilepsy. Neurotherapeutics.

[87] Yi X., Manickam D.S., Brynskikh A., Kabanov A.V. (2014). Agile delivery of protein therapeutics to CNS. J. Control. Release.

[88] Blasberg R., Patlak C., Fenstermacher J. (1975). Intrathecal chemotherapy: Brain tissue profiles after ventriculocisternal perfusion. J. Pharm. Exp. Ther..

[89] Chen G., Zhang X., Yang F., Mu L. (2010). Disposition of nanoparticle-based delivery system via inner ear administration. Curr. Drug Metab..

[90] Zhang X., Chen G., Wen L., Yang F., Shao A.-l., Li X., Long W., Mu L. (2013). Novel multiple agents loaded PLGA nanoparticles for brain delivery via inner ear administration: In vitro and in vivo evaluation. Eur. J. Pharm. Sci..

[91] Lochhead J.J., Thorne R.G. (2012). Intranasal delivery of biologics to the central nervous system. Adv. Drug Deliv. Rev..

[92] Zhang T.-T., Li W., Meng G., Wang P., Liao W. (2016). Strategies for transporting nanoparticles across the blood–brain barrier. Biomater. Sci..

[93] Bellavance M.-A., Blanchette M., Fortin D. (2008). Recent advances in blood–brain barrier disruption as a CNS delivery strategy. AAPS J..

[94] Wilson B. (2009). Brain targeting PBCA nanoparticles and the blood–brain barrier. Nanomedicine.

[95] Busquets M.A., Espargaró A., Sabaté R., Estelrich J. (2015). Magnetic nanoparticles cross the blood-brain barrier: When physics rises to a challenge. Nanomaterials.

[96] Holmes D. (2013). The next big things are tiny. Lancet Neurol..

[97] Re F., Gregori M., Masserini M. (2012). Nanotechnology for neurodegenerative disorders. Maturitas.

[98] Haque S., Md S., Alam M.I., Sahni J.K., Ali J., Baboota S. (2012). Nanostructure-based drug delivery systems for brain targeting. Drug Dev. Ind. Pharm..

[99] Wicki A., Witzigmann D., Balasubramanian V., Huwyler J. (2015). Nanomedicine in cancer therapy: Challenges, opportunities, and clinical applications. J. Control. Release.

[100] Malikmammadov E., Tanir T.E., Kiziltay A., Hasirci V., Hasirci N. (2018). PCL and PCL-based materials in biomedical applications. J. Biomater. Sci. Polym. Ed..

[101] Bregoli L., Movia D., Gavigan-Imedio J.D., Lysaght J., Reynolds J., Prina-Mello A. (2016). Nanomedicine applied to translational oncology: A future perspective on cancer treatment. Nanomed. Nanotechnol. Biol. Med..

[102] Truong N.P., Whittaker M.R., Mak C.W., Davis T.P. (2015). The importance of nanoparticle shape in cancer drug delivery. Expert Opin. Drug Deliv..

[103] Stylianopoulos T., Poh M.-Z., Insin N., Bawendi M.G., Fukumura D., Munn L.L., Jain R.K. (2010). Diffusion of particles in the extracellular matrix: The effect of repulsive electrostatic interactions. Biophys. J..

[104] Adabi M., Naghibzadeh M., Adabi M., Zarrinfard M.A., Esnaashari S.S., Seifalian A.M., Faridi-Majidi R., Tanimowo Aiyelabegan H., Ghanbari H. (2017). Biocompatibility and nanostructured materials: Applications in nanomedicine. Artif. Cells Nanomed. Biotechnol..

[105] Wong H.L., Wu X.Y., Bendayan R. (2012). Nanotechnological advances for the delivery of CNS therapeutics. Adv. Drug Deliv. Rev..

[106] Saraiva C., Praça C., Ferreira R., Santos T., Ferreira L., Bernardino L. (2016). Nanoparticle-mediated brain drug delivery: Overcoming blood–brain barrier to treat neurodegenerative diseases. J. Control. Release.

[107] Chapman C.D., Frey W.H., Craft S., Danielyan L., Hallschmid M., Schiöth H.B., Benedict C. (2013). Intranasal treatment of central nervous system dysfunction in humans. Pharm. Res..

[108] Koo Y.-E.L., Reddy G.R., Bhojani M., Schneider R., Philbert M.A., Rehemtulla A., Ross B.D., Kopelman R. (2006). Brain cancer diagnosis and therapy with nanoplatforms. Adv. Drug Deliv. Rev..

[109] Koffie R.M., Farrar C.T., Saidi L.-J., William C.M., Hyman B.T., Spires-Jones T.L. (2011). Nanoparticles enhance brain delivery of blood–brain barrier-impermeable probes for in vivo optical and magnetic resonance imaging. Proc. Natl. Acad. Sci. USA.

[110] Winer J.L., Kim P.E., Law M., Liu C.Y., Apuzzo M.L. (2011). Visualizing the future: Enhancing neuroimaging with nanotechnology. World Neurosurg..

[111] Kelkar S.S., Reineke T.M. (2011). Theranostics: Combining imaging and therapy. Bioconjug. Chem..

[112] Salvati A., Pitek A.S., Monopoli M.P., Prapainop K., Bombelli F.B., Hristov D.R., Kelly P.M., Åberg C., Mahon E., Dawson K.A. (2013). Transferrin-functionalized nanoparticles lose their targeting capabilities when a biomolecule corona adsorbs on the surface. Nat. Nanotechnol..

[113] Hadjidemetriou M., Al-Ahmady Z., Mazza M., Collins R.F., Dawson K., Kostarelos K. (2015). In vivo biomolecule corona around blood-circulating, clinically used and antibody-targeted lipid bilayer nanoscale vesicles. ACS Nano.

[114] Downs M.E., Buch A., Karakatsani M.E., Konofagou E.E., Ferrera V.P. (2015). Blood-brain barrier opening in behaving non-human primates via focused ultrasound with systemically administered microbubbles. Sci. Rep..

[115] Meyers J.D., Doane T., Burda C., Basilion J.P. (2013). Nanoparticles for imaging and treating brain cancer. Nanomedicine.

[116] Tian G., Yin W., Jin J., Zhang X., Xing G., Li S., Gu Z., Zhao Y. (2014). Engineered design of theranostic upconversion nanoparticles for tri-modal upconversion luminescence/magnetic resonance/X-ray computed tomography imaging and targeted delivery of combined anticancer drugs. J. Mater. Chem. B.

[117] Lollo G., Vincent M., Ullio-Gamboa G., Lemaire L., Franconi F., Couez D., Benoit J.-P. (2015). Development of multifunctional lipid nanocapsules for the co-delivery of paclitaxel and CpG-ODN in the treatment of glioblastoma. Int. J. Pharm..

[118] Allard E., Jarnet D., Vessières A., Vinchon-Petit S., Jaouen G., Benoit J.-P., Passirani C. (2010). Local delivery of ferrociphenol lipid nanocapsules followed by external radiotherapy as a synergistic treatment against intracranial 9L glioma xenograft. Pharm. Res..

[119] Fourniols T., Randolph L.D., Staub A., Vanvarenberg K., Leprince J.G., Préat V., des Rieux A., Danhier F. (2015). Temozolomide-loaded photopolymerizable PEG-DMA-based hydrogel for the treatment of glioblastoma. J. Control. Release.

[120] Huynh N.T., Passirani C., Allard-Vannier E., Lemaire L., Roux J., Garcion E., Vessieres A., Benoit J.-P. (2012). Administration-dependent efficacy of ferrociphenol lipid nanocapsules for the treatment of intracranial 9L rat gliosarcoma. Int. J. Pharm..

[121] Laine A.-L., Huynh N.T., Clavreul A., Balzeau J., Béjaud J., Vessieres A., Benoit J.-P., Eyer J., Passirani C. (2012). Brain tumour targeting strategies via coated ferrociphenol lipid nanocapsules. Eur. J. Pharm. Biopharm..

[122] Huynh N.T., Morille M., Bejaud J., Legras P., Vessieres A., Jaouen G., Benoit J.-P., Passirani C. (2011). Treatment of 9L gliosarcoma in rats by ferrociphenol-loaded lipid nanocapsules based on a passive targeting strategy via the EPR effect. Pharm. Res..

[123] Kumar A., Ahuja A., Ali J., Baboota S. (2016). Curcumin-loaded lipid nanocarrier for improving bioavailability, stability and cytotoxicity against malignant glioma cells. Drug Deliv..

[124] Verreault M., Wehbe M., Strutt D., Masin D., Anantha M., Walker D., Chu F., Backstrom I., Kalra J., Waterhouse D. (2015). Determination of an optimal dosing schedule for combining Irinophore C™ and temozolomide in an orthotopic model of glioblastoma. J. Control. Release.

[125] G Gritsenko P., Ilina O., Friedl P. (2012). Interstitial guidance of cancer invasion. J. Pathol..

[126] Pluen A., Boucher Y., Ramanujan S., McKee T.D., Gohongi T., di Tomaso E., Brown E.B., Izumi Y., Campbell R.B., Berk D.A. (2001). Role of tumor–host interactions in interstitial diffusion of macromolecules: Cranial vs. subcutaneous tumors. Proc. Natl. Acad. Sci. USA.

[127] Gelperina S., Maksimenko O., Khalansky A., Vanchugova L., Shipulo E., Abbasova K., Berdiev R., Wohlfart S., Chepurnova N., Kreuter J. (2010). Drug delivery to the brain using surfactant-coated poly (lactide-co-glycolide) nanoparticles: Influence of the formulation parameters. Eur. J. Pharm. Biopharm..

[128] Garcia-Garcia E., Andrieux K., Gil S., Couvreur P. (2005). Colloidal carriers and blood–brain barrier (BBB) translocation: A way to deliver drugs to the brain?. Int. J. Pharm..

[129] Esmaeili F., Ghahremani M.H., Esmaeili B., Khoshayand M.R., Atyabi F., Dinarvand R. (2008). PLGA nanoparticles of different surface properties: Preparation and evaluation of their body distribution. Int. J. Pharm..

[130] Hermanson G.T. (2013). Bioconjugate Techniques.

[131] Nobs L., Buchegger F., Gurny R., Allémann E. (2004). Current methods for attaching targeting ligands to liposomes and nanoparticles. J. Pharm. Sci..

[132] Koo B.-K., Lim H.-S., Song R., Yoon M.-J., Yoon K.-J., Moon J.-S., Kim Y.-W., Kwon M.-c., Yoo K.-W., Kong M.-P. (2005). Mind bomb 1 is essential for generating functional Notch ligands to activate Notch. Development.

[133] Webster R., Didier E., Harris P., Siegel N., Stadler J., Tilbury L., Smith D. (2007). PEGylated proteins: Evaluation of their safety in the absence of definitive metabolism studies. Drug Metab. Dispos..

[134] Jevševar S., Kunstelj M., Porekar V.G. (2010). PEGylation of therapeutic proteins. Biotechnol. J. Healthc. Nutr. Technol..

[135] Koziara J.M., Lockman P.R., Allen D.D., Mumper R.J. (2004). Paclitaxel nanoparticles for the potential treatment of brain tumors. J. Control. Release.

[136] Petri B., Bootz A., Khalansky A., Hekmatara T., Müller R., Uhl R., Kreuter J., Gelperina S. (2007). Chemotherapy of brain tumour using doxorubicin bound to surfactant-coated poly (butyl cyanoacrylate) nanoparticles: Revisiting the role of surfactants. J. Control. Release.

[137] Steiniger S.C., Kreuter J., Khalansky A.S., Skidan I.N., Bobruskin A.I., Smirnova Z.S., Severin S.E., Uhl R., Kock M., Geiger K.D. (2004). Chemotherapy of glioblastoma in rats using doxorubicin-loaded nanoparticles. Int. J. Cancer.

[138] Hekmatara T., Bernreuther C., Khalansky A., Theisen A., Weissenberger J., Matschke J., Gelperina S., Kreuter J., Glatzel M. (2009). Efficient systemic therapy of rat glioblastoma by nanoparticle-bound doxorubicin is due to antiangiogenic effects. Clin. Neuropathol..

[139] Kuo Y.-C., Chen Y.-C. (2015). Targeting delivery of etoposide to inhibit the growth of human glioblastoma multiforme using lactoferrin-and folic acid-grafted poly (lactide-co-glycolide) nanoparticles. International J. Pharm..

[140] Labhasetwar V., Song C., Humphrey W., Shebuski R., Levy R.J. (1998). Arterial uptake of biodegradable nanoparticles: Effect of surface modifications. J. Pharm. Sci..

[141] Zhang D., Mehler M.F., Song Q., Kessler J.A. (1998). Development of bone morphogenetic protein receptors in the nervous system and possible roles in regulating trkC expression. J. Neurosci..

[142] Huang R., Ke W., Han L., Liu Y., Shao K., Ye L., Lou J., Jiang C., Pei Y. (2009). Brain-targeting mechanisms of lactoferrin-modified DNA-loaded nanoparticles. J. Cereb. Blood Flow Metab..

[143] Zhang B., Sun X., Mei H., Wang Y., Liao Z., Chen J., Zhang Q., Hu Y., Pang Z., Jiang X. (2013). LDLR-mediated peptide-22-conjugated nanoparticles for dual-targeting therapy of brain glioma. Biomaterials.

[144] Gao H., Zhang S., Cao S., Yang Z., Pang Z., Jiang X. (2014). Angiopep-2 and activatable cell-penetrating peptide dual-functionalized nanoparticles for systemic glioma-targeting delivery. Mol. Pharm..

[145] Zhang B., Wang H., Liao Z., Wang Y., Hu Y., Yang J., Shen S., Chen J., Mei H., Shi W. (2014). EGFP–EGF1-conjugated nanoparticles for targeting both neovascular and glioma cells in therapy of brain glioma. Biomaterials.

[146] Callewaert M., Dukic S., Van Gulick L., Vittier M., Gafa V., Andry M.C., Molinari M., Roullin V.G. (2013). Etoposide encapsulation in surface-modified poly (lactide-co-glycolide) nanoparticles strongly enhances glioma antitumor efficiency. J. Biomed. Mater. Res. Part A.

[147] Dhami N.K., Pandey R.S., Jain U.K., Chandra R., Madan J. (2014). Non-aggregated protamine-coated poly (lactide-co-glycolide) nanoparticles of cisplatin crossed blood–brain barrier, enhanced drug delivery and improved therapeutic index in glioblastoma cells: In vitro studies. J. Microencapsul..

[148] Peer D., Karp J.M., Hong S., Farokhzad O.C., Margalit R., Langer R. (2007). Nanocarriers as an emerging platform for cancer therapy. Nat. Nanotechnol..

[149] Liu Z., Robinson J.T., Tabakman S.M., Yang K., Dai H. (2011). Carbon materials for drug delivery & cancer therapy. Mater. Today.

[150] West K.R., Otto S. (2005). Reversible covalent chemistry in drug delivery. Curr. Drug Discov. Technol..

[151] Reis C.P., Neufeld R.J., Ribeiro A.J., Veiga F. (2006). Nanoencapsulation I. Methods for preparation of drug-loaded polymeric nanoparticles. Nanomed. Nanotechnol. Biol. Med..

[152] Béduneau A., Saulnier P., Benoit J.-P. (2007). Active targeting of brain tumors using nanocarriers. Biomaterials.

[153] Crotts G., Park T.G. (1998). Protein delivery from poly (lactic-co-glycolic acid) biodegradable microspheres: Release kinetics and stability issues. J. Microencapsul..

[154] Shi J., Xiao Z., Kamaly N., Farokhzad O.C. (2011). Self-assembled targeted nanoparticles: Evolution of technologies and bench to bedside translation. Acc. Chem. Res..

[155] Davis M.E. (2009). The first targeted delivery of siRNA in humans via a self-assembling, cyclodextrin polymer-based nanoparticle: From concept to clinic. Mol. Pharm..

[156] Drummond D.C., Meyer O., Hong K., Kirpotin D.B., Papahadjopoulos D. (1999). Optimizing liposomes for delivery of chemotherapeutic agents to solid tumors. Pharm. Rev..

[157] Masood F. (2016). Polymeric nanoparticles for targeted drug delivery system for cancer therapy. Mater. Sci. Eng. C.

[158] Kreuter J., Alyautdin R.N., Kharkevich D.A., Ivanov A.A. (1995). Passage of peptides through the blood-brain barrier with colloidal polymer particles (nanoparticles). Brain Res..

[159] Calvo P., Gouritin B., Chacun H., Desmaële D., D’Angelo J., Noel J.-P., Georgin D., Fattal E., Andreux J.P., Couvreur P. (2001). Long-circulating PEGylated polycyanoacrylate nanoparticles as new drug carrier for brain delivery. Pharm. Res..

[160] Gao X., Tao W., Lu W., Zhang Q., Zhang Y., Jiang X., Fu S. (2006). Lectin-conjugated PEG–PLA nanoparticles: Preparation and brain delivery after intranasal administration. Biomaterials.

[161] Kamaly N., Xiao Z., Valencia P.M., Radovic-Moreno A.F., Farokhzad O.C. (2012). Targeted polymeric therapeutic nanoparticles: Design, development and clinical translation. Chem. Soc. Rev..

[162] Sugano M., Egilmez N.K., Yokota S.J., Chen F.-A., Harding J., Huang S.K., Bankert R.B. (2000). Antibody targeting of doxorubicin-loaded liposomes suppresses the growth and metastatic spread of established human lung tumor xenografts in severe combined immunodeficient mice. Cancer Res..

[163] Alexis F., Pridgen E., Molnar L.K., Farokhzad O.C. (2008). Factors affecting the clearance and biodistribution of polymeric nanoparticles. Mol. Pharm..

[164] Kirpotin D.B., Drummond D.C., Shao Y., Shalaby M.R., Hong K., Nielsen U.B., Marks J.D., Benz C.C., Park J.W. (2006). Antibody targeting of long-circulating lipidic nanoparticles does not increase tumor localization but does increase internalization in animal models. Cancer Res..

[165] Bartlett D.W., Su H., Hildebrandt I.J., Weber W.A., Davis M.E. (2007). Impact of tumor-specific targeting on the biodistribution and efficacy of siRNA nanoparticles measured by multimodality in vivo imaging. Proc. Natl. Acad. Sci. USA.

[166] Jain A., Jain A., Garg N.K., Tyagi R.K., Singh B., Katare O.P., Webster T.J., Soni V. (2015). Surface engineered polymeric nanocarriers mediate the delivery of transferrin–methotrexate conjugates for an improved understanding of brain cancer. Acta Biomater..

[167] Chang J., Jallouli Y., Kroubi M., Yuan X.-B., Feng W., Kang C.-S., Pu P.-Y., Betbeder D. (2009). Characterization of endocytosis of transferrin-coated PLGA nanoparticles by the blood–brain barrier. Int. J. Pharm..

[168] Ding H., Inoue S., Ljubimov A.V., Patil R., Portilla-Arias J., Hu J., Konda B., Wawrowsky K.A., Fujita M., Karabalin N. (2010). Inhibition of brain tumor growth by intravenous poly (β-L-malic acid) nanobioconjugate with pH-dependent drug release. Proc. Natl. Acad. Sci. USA.

[169] Xin H., Jiang X., Gu J., Sha X., Chen L., Law K., Chen Y., Wang X., Jiang Y., Fang X. (2011). Angiopep-conjugated poly (ethylene glycol)-co-poly (ε-caprolactone) nanoparticles as dual-targeting drug delivery system for brain glioma. Biomaterials.

[170] Li J., Feng L., Fan L., Zha Y., Guo L., Zhang Q., Chen J., Pang Z., Wang Y., Jiang X. (2011). Targeting the brain with PEG–PLGA nanoparticles modified with phage-displayed peptides. Biomaterials.

[171] Xin H., Sha X., Jiang X., Zhang W., Chen L., Fang X. (2012). Anti-glioblastoma efficacy and safety of paclitaxel-loading Angiopep-conjugated dual targeting PEG-PCL nanoparticles. Biomaterials.

[172] Geldenhuys W., Wehrung D., Groshev A., Hirani A., Sutariya V. (2015). Brain-targeted delivery of doxorubicin using glutathione-coated nanoparticles for brain cancers. Pharm. Dev. Technol..

[173] Fornaguera C., Dols-Perez A., Caldero G., Garcia-Celma M., Camarasa J., Solans C. (2015). PLGA nanoparticles prepared by nano-emulsion templating using low-energy methods as efficient nanocarriers for drug delivery across the blood–brain barrier. J. Control. Release.

[174] Cui Y., Zhang M., Zeng F., Jin H., Xu Q., Huang Y. (2016). Dual-targeting magnetic PLGA nanoparticles for codelivery of paclitaxel and curcumin for brain tumor therapy. ACS Appl. Mater. Interfaces.

[175] Malinovskaya Y., Melnikov P., Baklaushev V., Gabashvili A., Osipova N., Mantrov S., Ermolenko Y., Maksimenko O., Gorshkova M., Balabanyan V. (2017). Delivery of doxorubicin-loaded PLGA nanoparticles into U87 human glioblastoma cells. Int. J. Pharm..

[176] Ramalho M., Sevin E., Gosselet F., Lima J., Coelho M., Loureiro J., Pereira M. (2018). Receptor-mediated PLGA nanoparticles for glioblastoma multiforme treatment. Int. J. Pharm..

[177] Ganipineni L.P., Ucakar B., Joudiou N., Riva R., Jérôme C., Gallez B., Danhier F., Préat V. (2019). Paclitaxel-loaded multifunctional nanoparticles for the targeted treatment of glioblastoma. J. Drug Target..

[178] Georgieva J., Hoekstra D., Zuhorn I. (2014). Smuggling drugs into the brain: An overview of ligands targeting transcytosis for drug delivery across the blood–brain barrier. Pharmaceutics.

[179] Chen Y., Liu L. (2012). Modern methods for delivery of drugs across the blood–brain barrier. Adv. Drug Deliv. Rev..

[180] Loureiro J.A., Gomes B., Coelho M.A., Carmo Pereira M.d., Rocha S. (2014). Targeting nanoparticles across the blood–brain barrier with monoclonal antibodies. Nanomedicine.

[181] Calzolari A., Larocca L.M., Deaglio S., Finisguerra V., Boe A., Raggi C., Ricci-Vitani L., Pierconti F., Malavasi F., De Maria R. (2010). Transferrin receptor 2 is frequently and highly expressed in glioblastomas. Transl. Oncol..

[182] Lee H.J., Engelhardt B., Lesley J., Bickel U., Pardridge W.M. (2000). Targeting rat anti-mouse transferrin receptor monoclonal antibodies through blood-brain barrier in mouse. J. Pharm. Exp. Ther..

[183] Schröder U., Sabel B.A. (1996). Nanoparticles, a drug carrier system to pass the blood-brain barrier, permit central analgesic effects of iv dalargin injections. Brain Res..

[184] Wohlfart S., Khalansky A.S., Gelperina S., Maksimenko O., Bernreuther C., Glatzel M., Kreuter J. (2011). Efficient chemotherapy of rat glioblastoma using doxorubicin-loaded PLGA nanoparticles with different stabilizers. PLoS ONE.

[185] Demeule M., Regina A., Che C., Poirier J., Nguyen T., Gabathuler R., Castaigne J.-P., Beliveau R. (2008). Identification and design of peptides as a new drug delivery system for the brain. J. Pharm. Exp. Ther..

[186] Demeule M., Currie J.C., Bertrand Y., Ché C., Nguyen T., Régina A., Gabathuler R., Castaigne J.P., Béliveau R. (2008). Involvement of the low-density lipoprotein receptor-related protein in the transcytosis of the brain delivery vector Angiopep-2. J. Neurochem..

[187] Shao K., Huang R., Li J., Han L., Ye L., Lou J., Jiang C. (2010). Angiopep-2 modified PE-PEG based polymeric micelles for amphotericin B delivery targeted to the brain. J. Control. Release.

[188] von Roemeling C., Jiang W., Chan C.K., Weissman I.L., Kim B.Y. (2017). Breaking down the barriers to precision cancer nanomedicine. Trends Biotechnol..

[189] Miele E., Spinelli G.P., Miele E., Tomao F., Tomao S. (2009). Albumin-bound formulation of paclitaxel (Abraxane® ABI-007) in the treatment of breast cancer. Int. J. Nanomed..

[190] Choi H.S., Liu W., Misra P., Tanaka E., Zimmer J.P., Ipe B.I., Bawendi M.G., Frangioni J.V. (2007). Renal clearance of quantum dots. Nat. Biotechnol..

[191] Lee J.E., Kim M.G., Jang Y.L., Lee M.S., Kim N.W., Yin Y., Lee J.H., Lim S.Y., Park J.W., Kim J. (2018). Self-assembled PEGylated albumin nanoparticles (SPAN) as a platform for cancer chemotherapy and imaging. Drug Deliv..

[192] Ruggiero A., Villa C.H., Bander E., Rey D.A., Bergkvist M., Batt C.A., Manova-Todorova K., Deen W.M., Scheinberg D.A., McDevitt M.R. (2010). Paradoxical glomerular filtration of carbon nanotubes. Proc. Natl. Acad. Sci. USA.

[193] Liu J., Yu M., Zhou C., Yang S., Ning X., Zheng J. (2013). Passive tumor targeting of renal-clearable luminescent gold nanoparticles: Long tumor retention and fast normal tissue clearance. J. Am. Chem. Soc..

[194] Blanco E., Shen H., Ferrari M. (2015). Principles of nanoparticle design for overcoming biological barriers to drug delivery. Nat. Biotechnol..

[195] Boucher Y., Baxter L.T., Jain R.K. (1990). Interstitial pressure gradients in tissue-isolated and subcutaneous tumors: Implications for therapy. Cancer Res..

[196] Jain R.K. (2005). Normalization of tumor vasculature: An emerging concept in antiangiogenic therapy. Science.

[197] Batchelor T.T., Sorensen A.G., di Tomaso E., Zhang W.-T., Duda D.G., Cohen K.S., Kozak K.R., Cahill D.P., Chen P.-J., Zhu M. (2007). AZD2171, a pan-VEGF receptor tyrosine kinase inhibitor, normalizes tumor vasculature and alleviates edema in glioblastoma patients. Cancer Cell.

[198] Jiang W., Huang Y., An Y., Kim B.Y. (2015). Remodeling tumor vasculature to enhance delivery of intermediate-sized nanoparticles. ACS Nano.

[199] Alexandrakis G., Brown E.B., Tong R.T., McKee T.D., Campbell R.B., Boucher Y., Jain R.K. (2004). Two-photon fluorescence correlation microscopy reveals the two-phase nature of transport in tumors. Nat. Med..

[200] McKee T.D., Grandi P., Mok W., Alexandrakis G., Insin N., Zimmer J.P., Bawendi M.G., Boucher Y., Breakefield X.O., Jain R.K. (2006). Degradation of fibrillar collagen in a human melanoma xenograft improves the efficacy of an oncolytic herpes simplex virus vector. Cancer Res..

[201] Cabral H., Matsumoto Y., Mizuno K., Chen Q., Murakami M., Kimura M., Terada Y., Kano M., Miyazono K., Uesaka M. (2011). Accumulation of sub-100 nm polymeric micelles in poorly permeable tumours depends on size. Nat. Nanotechnol..

[202] Ferrari M. (2010). Frontiers in cancer nanomedicine: Directing mass transport through biological barriers. Trends Biotechnol..

[203] Wong C., Stylianopoulos T., Cui J., Martin J., Chauhan V.P., Jiang W., Popović Z., Jain R.K., Bawendi M.G., Fukumura D. (2011). Multistage nanoparticle delivery system for deep penetration into tumor tissue. Proc. Natl. Acad. Sci. USA.

[204] Huang C.-F., Yao G.-H., Liang R.-P., Qiu J.-D. (2013). Graphene oxide and dextran capped gold nanoparticles based surface plasmon resonance sensor for sensitive detection of concanavalin A. Biosens. Bioelectron..

[205] Szakács G., Paterson J.K., Ludwig J.A., Booth-Genthe C., Gottesman M.M. (2006). Targeting multidrug resistance in cancer. Nat. Rev. Drug Discov..

[206] Aller S.G., Yu J., Ward A., Weng Y., Chittaboina S., Zhuo R., Harrell P.M., Trinh Y.T., Zhang Q., Urbatsch I.L. (2009). Structure of P-glycoprotein reveals a molecular basis for poly-specific drug binding. Science.

[207] Gottesman M.M., Fojo T., Bates S.E. (2002). Multidrug resistance in cancer: Role of ATP–dependent transporters. Nat. Rev. Cancer.

[208] Fletcher J.I., Haber M., Henderson M.J., Norris M.D. (2010). ABC transporters in cancer: More than just drug efflux pumps. Nat. Rev. Cancer.

[209] Dean M., Fojo T., Bates S. (2005). Tumour stem cells and drug resistance. Nat. Rev. Cancer.

[210] Wu J., Lee A., Lu Y., Lee R.J. (2007). Vascular targeting of doxorubicin using cationic liposomes. Int. J. Pharm..

[211] Wong H.L., Bendayan R., Rauth A.M., Xue H.Y., Babakhanian K., Wu X.Y. (2006). A mechanistic study of enhanced doxorubicin uptake and retention in multidrug resistant breast cancer cells using a polymer-lipid hybrid nanoparticle system. J. Pharm. Exp. Ther..

[212] Xiong X.-B., Lavasanifar A. (2011). Traceable multifunctional micellar nanocarriers for cancer-targeted co-delivery of MDR-1 siRNA and doxorubicin. ACS Nano.

[213] Anselmo A.C., Mitragotri S. (2016). Nanoparticles in the clinic. Bioeng. Transl. Med..

[214] Dobrovolskaia M.A., Aggarwal P., Hall J.B., McNeil S.E. (2008). Preclinical studies to understand nanoparticle interaction with the immune system and its potential effects on nanoparticle biodistribution. Mol. Pharm..

[215] Moghimi S.M., Hunter A.C., Murray J.C. (2001). Long-circulating and target-specific nanoparticles: Theory to practice. Pharm. Rev..

[216] Barenholz Y.C. (2012). Doxil®—The first FDA-approved nano-drug: Lessons learned. J. Control. Release.

[217] Carnevale J., Ko A.H. (2016). MM-398 (nanoliposomal irinotecan): Emergence of a novel therapy for the treatment of advanced pancreatic cancer. Future Oncol..

[218] Chang T., Shiah H., Yang C., Yeh K., Cheng A., Shen B., Wang Y., Yeh C., Chiang N., Chang J.-Y. (2015). Phase I study of nanoliposomal irinotecan (PEP02) in advanced solid tumor patients. Cancer Chemother. Pharmacol..

[219] Kreuter J. (1978). Nanoparticles and nanocapsules—New dosage forms in the nanometer size range. Pharm. Acta Helv..

[220] Valle J.W., Armstrong A., Newman C., Alakhov V., Pietrzynski G., Brewer J., Campbell S., Corrie P., Rowinsky E.K., Ranson M. (2011). A phase 2 study of SP1049C, doxorubicin in P-glycoprotein-targeting pluronics, in patients with advanced adenocarcinoma of the esophagus and gastroesophageal junction. Investig. New Drugs.

[221] Andrade F., Almeida A., Rafael D., Schwartz S., Sarmento B. (2018). Micellar-Based Nanoparticles for Cancer Therapy and Bioimaging. Nanooncology.

[222] Matsumura Y., Hamaguchi T., Ura T., Muro K., Yamada Y., Shimada Y., Shirao K., Okusaka T., Ueno H., Ikeda M. (2004). Phase I clinical trial and pharmacokinetic evaluation of NK911, a micelle-encapsulated doxorubicin. Br. J. Cancer.

[223] Ahn H.K., Jung M., Sym S.J., Shin D.B., Kang S.M., Kyung S.Y., Park J.-W., Jeong S.H., Cho E.K. (2014). A phase II trial of Cremorphor EL-free paclitaxel (Genexol-PM) and gemcitabine in patients with advanced non-small cell lung cancer. Cancer Chemother. Pharmacol..

[224] Kim T.-Y., Kim D.-W., Chung J.-Y., Shin S.G., Kim S.-C., Heo D.S., Kim N.K., Bang Y.-J. (2004). Phase I and pharmacokinetic study of Genexol-PM, a cremophor-free, polymeric micelle-formulated paclitaxel, in patients with advanced malignancies. Clin. Cancer Res..

[225] Autio K.A., Dreicer R., Anderson J., Garcia J.A., Alva A., Hart L.L., Milowsky M.I., Posadas E.M., Ryan C.J., Graf R.P. (2018). Safety and efficacy of BIND-014, a docetaxel nanoparticle targeting prostate-specific membrane antigen for patients with metastatic castration-resistant prostate cancer: A phase 2 clinical trial. JAMA Oncol..

[226] Chakraborty C., Sharma A.R., Sharma G., Doss C.G.P., Lee S.-S. (2017). Therapeutic miRNA and siRNA: Moving from bench to clinic as next generation medicine. Mol. Therapy-Nucleic Acids.

[227] Giri V.P., Kanodia S., Giri O.P., Sumit K. (2017). Anti-nicotine vaccine: Current status. Int. J. Basic Clin. Pharm..

[228] Gu F., Zhang L., Teply B.A., Mann N., Wang A., Radovic-Moreno A.F., Langer R., Farokhzad O.C. (2008). Precise engineering of targeted nanoparticles by using self-assembled biointegrated block copolymers. Proc. Natl. Acad. Sci. USA.

